# A Systems Biology Approach Reveals Differences in the Dynamics of Colonization and Degradation of Grass vs. Hay by Rumen Microbes with Minor Effects of Vitamin E Supplementation

**DOI:** 10.3389/fmicb.2017.01456

**Published:** 2017-08-03

**Authors:** Alejandro Belanche, Charles J. Newbold, Wanchang Lin, Pauline Rees Stevens, Alison H. Kingston-Smith

**Affiliations:** ^1^Institute of Biological, Environmental and Rural Sciences, Aberystwyth University Aberystwyth, United Kingdom; ^2^Estacion Experimental del Zaidín, Consejo Superior de Investigaciones Científicas Granada, Spain

**Keywords:** colonization, grass, hay, rumen fermentation, rumen microbiome, plant degradation, vitamin E

## Abstract

Increasing the efficiency of utilization of fresh and preserved forage is a key target for ruminant science. Vitamin E is often used as additive to improve product quality but its impact of the rumen function is unknown. This study investigated the successional microbial colonization of ryegrass (GRA) vs. ryegrass hay (HAY) in presence of zero or 50 IU/d supplementary vitamin E, using a rumen simulation technique. A holistic approach was used to link the dynamics of feed degradation with the structure of the liquid-associated (LAB) and solid-associated bacteria (SAB). Results showed that forage colonization by SAB was a tri-phasic process highly affected by the forage conservation method: Early colonization (0–2 h after feeding) by rumen microbes was 2× faster for GRA than HAY diets and dominated by *Lactobacillus* and *Prevotella* which promoted increased levels of lactate (+56%) and ammonia (+18%). HAY diets had lower DM degradation (-72%) during this interval being *Streptococcus* particularly abundant. During secondary colonization (4–8 h) the SAB community increased in size and decreased in diversity as the secondary colonizers took over (*Pseudobutyrivibrio*) promoting the biggest differences in the metabolomics profile between diets. Secondary colonization was 3× slower for HAY vs. GRA diets, but this delay was compensated by a greater bacterial diversity (+197 OTUs) and network complexity resulting in similar feed degradations. Tertiary colonization (>8 h) consisted of a slowdown in the colonization process and simplification of the bacterial network. This slowdown was less evident for HAY diets which had higher levels of tertiary colonizers (*Butyrivibrio* and *Ruminococcus*) and may explain the higher DM degradation (+52%) during this interval. The LAB community was particularly active during the early fermentation of GRA and during the late fermentation for HAY diets indicating that the availability of nutrients in the liquid phase reflects the dynamics of feed degradation. Vitamin E supplementation had minor effects but promoted a simplification of the LAB community and a slight acceleration in the SAB colonization sequence which could explain the higher DM degradation during the secondary colonization. Our findings suggest that when possible, grass should be fed instead of hay, in order to accelerate feed utilization by rumen microbes.

## Introduction

The rumen microbiota has been described as “the most elegant and highly evolved cellulose-digesting system in nature” (Weimer et al., 2009). As a result ruminants are the only livestock which potentially do not compete for human edible foods (Gill et al., 2010). Traditional grazing systems generally have a positive impact on fatty acid profile in meat and milk (Mohammed et al., 2009), animal health and welfare, farm profitability (Kennedy et al., 2005) and a positive perception in society. However, in the context of a growing human population and demand for animal products, modern ruminant production systems based on large-scale farms tend to replace fresh pastures by preserved forages such as hay in order to allow a greater control of the diet (Van den Pol-van Dasselaar et al., 2008). Nevertheless, this decision is often arbitrary based on farm management concerns, without taking in to consideration the impact of the feeding strategy on rumen microbiota, feed efficiency and on the environment (Van den Pol-van Dasselaar et al., 2008).

Several studies have described strategies to maximize forage quality (Minson, 1990) and described the main differences between fresh grass and hay in terms of rumen digestion of nutrients using cannulated animals (Petit and Tremblay, 1992; Holden et al., 1994). More recent studies based on molecular techniques have defined the bacterial colonization pattern for fresh grass (Edwards et al., 2007; Huws et al., 2013, 2016; Mayorga et al., 2016) and grass hay (Piao et al., 2014) but no direct comparison between grass and hay has been reported. These studies focused only on the feed colonization by solid-associated microbes which under ordinary feeding conditions account for 75% of the microbial matter in the rumen (Forsberg and Lam, 1977; Craig et al., 1987) and are responsible for most of the endoglucanase (90%), xylanase (88%), amylase (70%) and protease activity (75%) in the rumen (McAllister et al., 1994a) as well as cellulose and hemicellulose (Williams and Strachan, 1984). However, the microbial subpopulation associated with the ruminal fluid represents 20–30% of the total microbes and is a mixture of microorganism that have detached from feed particles as well as those that subsist on soluble feed components (Forsberg and Lam, 1977; Craig et al., 1987). This community has little direct involvement in the digestion of insoluble feed particles but represents an integral part of the ruminal ecosystem because it is the first microbial community to interact with the newly ingested substrate (McAllister et al., 1994b). Thus, further studies integrating a detailed description of the liquid- (LAB) and solid-associated bacteria (SAB) are needed to fully understand the dynamics of forage colonization and utilization by rumen microbes.

Fresh grass, in contrast to preserved forages, is naturally rich in antioxidants, such as vitamin E, which should meet animal requirements (National Research Council [NRC], 2001). However, there is an increasing tendency to include supra-nutritional levels of synthetic vitamin E (α-tocopheryl acetate) in ruminant diets to prevent lipid oxidation in milk and improve color stability and the shelf life of red meat (Arnold et al., 1992). It has also been suggested, that high levels of vitamin E supplementation (above 8,000 IU/d per cow) can help to prevent the milk fat depression typically observed with low-fiber diets (Focant et al., 1998) as well as to increase milk fat content by 6% (Kay et al., 2005). However, it is still unclear whether these outcomes rely on a direct vitamin E effect on the rumen fat bio-hydrogenation pattern, or on an indirect effect mediated by a shift in the rumen microbiota which could favor feed digestion. The rumen is considered to be an anaerobic environment, but dissolved oxygen can be present at concentrations as high as 3 mmol/L for at least 18 h during the day (Stewart and Bryant, 1988) promoting oxidation of cellular membranes and accumulation of free radicals (Naziroglu et al., 2002). As facultative and obligate anaerobes, the rumen bacteria, protozoa, methanogens and fungi show different abilities to grow in the presence of oxygen (Lloyd et al., 1989), with the fibrolytic bacteria being particularly oxygen sensitive (Loesche, 1969). Thus, supplementation with antioxidants such as vitamin E might theoretically favor the proliferation of strictly anaerobic microbes (Van Soest, 1994) and improve feed digestibility and nutrient utilization efficiency which are considered key targets for ruminant science. In a recent study we noted that vitamin E supplementation promoted a moderate increase (+8%) in feed digestibility (Belanche et al., 2016a). Moreover, we demonstrated that despite having similar chemical composition, grass diets had a positive effect on bacterial protein synthesis (+16%) whilst grass hay increased methane emissions (+35%), suggesting that substantial differences in the feed colonization and utilization pattern must have occurred.

This study aims to investigate the differences in the rumen microbiome between fresh grass (GRA) and grass hay (HAY) diets when fed alone or supplemented by vitamin E. It was hypothesized that vitamin E supplementation could promote a shift in the rumen microbiota and have a positive impact on the feed colonization process, being this effect diet dependent based on its natural vitamin E content. A holistic approach based on systems biology was adopted to reveal the mode of action of these feeding strategies based on linking rumen function and the microbiome in a rumen simulation technique (Rusitec). This approach described the dynamics of feed colonization, degradation and utilization using complementary techniques (isotope labeling, quantitative PCR and metabolomics). Moreover, 16SrRNA (cDNA) Next Generation Sequencing of LAB and SAB communities was used to characterize time-related changes in diversity, abundances and bacterial networks. By basing this experiment on RNA rather than DNA, these data provide an insight specifically into the metabolically active rumen microbiome and its role on rumen function, making this study among the most comprehensive of its type to date.

## Materials and Methods

### Treatments and Forage Collection

Animal trials were conducted in accordance to the Home Office Scientific Procedures, Act 1986 (PLL 40/3653; PIL 40/9798) and protocols were approved by the Aberystwyth University Ethical Committee. A 2 × 2 factorial arrangement of treatments was conducted with two types of forage [grass (**GRA**) vs. grass hay (**HAY**)] and two levels of vitamin E supplementation [non-supplemented (**-**) vs. supplemented (**+**)] giving four treatments (GRA-, GRA+, HAY-, HAY+). A commercial dl-α-tocopheryl acetate with 50% silica adsorbate (Frank Wright Trouw Nutrition, Ashbourne, United Kingdom) was mixed with the concentrate and dosed at 50 IU/d based on the minimum effective dose observed in a previous study (Belanche et al., 2016a). The experimental diets had 80:20 forage to concentrate ratio and their composition is described in Supplementary Table 1. Forage was obtained from a 3rd cut of a ryegrass monoculture (*Lolium perenne* L. cv. AberMagic, Germinal GB Ltd, Lincoln, United Kingdom). Grass was cut at 5 cm above soil level and 20 kg of fresh grass was immediately frozen and kept at -20°C for GRA treatment, while a further 20 kg were left to dry in the field for 48 h and finished in an air force oven at 25°C for 5 days to generate HAY treatment. Both forages were chopped to between 2 and 4 cm length by passing through a garden shredder (Bosch AXT Rapid 2200, United Kingdom) and concentrate was ground to 1 mm^2^ using a hammer mill.

### Rumen Simulation Technique

The incubation procedure was performed as previously described (Belanche et al., 2016a). Briefly, a total of 16 Rusitec vessels, which were considered as experimental units, were used in a single incubation period. Experimental diets were randomly allocated to the vessels and inoculated with rumen fluid from four rumen-cannulated cows fed ryegrass hay and concentrate at 80:20 on DM basis. Rumen content from each cow was sampled before morning feeding, filtered through a double layer of muslin, diluted 1:1 with an anaerobic buffer solution (McDougall, 1948), and dispensed into four vessels receiving one treatment each (800 mL effective volume). Vessels were kept at 39°C under permanent vertical agitation and continuously infused with artificial saliva (McDougall, 1948) at a dilution rate of 3.35% h^-1^. Feed (11.25g DM/d) was placed in nylon bags (200 mm × 80 mm, pore size 100 μm^2^) and incubated in each vessel for 48 h, thus two feed bags were present in each vessel at any time. After incubation bags were squeezed, washed with 50 ml of artificial saliva and the liquid fraction of the washing was returned to the vessels before adding a new bag.

The incubation trial consisted of 18 days, using the first 14 days for adaptation and the last 4 for sampling. During the sampling period (days 15, 16, and 17) fermenters were fed with three small nylon bags (100 mm × 50 mm, pore size 100 μm^2^) each containing 2 g DM and one conventional bag containing 9 g DM. To study the colonization dynamics, plant residues were collected after incubation for 2, 4, 8, and 48 h, respectively. Plant residues were rinsed twice to remove the loosely associated microbes; first with 25 mL of artificial saliva which was returned to the vessel and secondly with distilled water (25 mL). The plant residues were then snap frozen in liquid N and pooled per vessel and time point and ground in liquid N. One fraction was used for RNA extraction, while the rest was freeze dried for chemical analysis and ^15^N determination. Fermentation pattern was described at the same time points in terms of vessel pH and redox potential, moreover vessel contents (25 mL) were sampled and divided in five sub-samples: VFA, ammonia and lactate samples were analyzed as previously described (Belanche et al., 2016b). A subsample (1.6 mL) was snap frozen and pooled per vessel and time-point for RNA extraction. For metabolomics one last sample (10 mL) was taken and separated into two fractions (Kingston-Smith et al., 2013): the cell free fraction was obtained from the supernatant (0.5 mL) after centrifugation at 10,000 × *g* for 15 min, while the bacteria in the planktonic phase (referred to as the pellet) was obtained from the sediment after removing the remaining supernatant. This pellet was then washed (twice) with 0.5 mL of distilled water and centrifugation was repeated. Both fractions were kept frozen until further analysis.

The kinetics of plant colonization by rumen microbes were investigated using two methods: ^15^N bacterial labeling and quantitative PCR. On day 10 vessels were infused with 3 mg/vessel of ^15^N, as (^15^NH_4_)_2_SO_4_ to label the ammonia-N pool. To label the microbial protein, from day 10 onwards ^15^N was added into the artificial saliva (3.7 mg/L). Plant residues from days 15, 16, and 17 were pooled by time point prior to RNA and ^15^N determination. On the last day of the experiment (d18) a SAB pellet was isolated from the 24 h plant residue. Rinsed plant residue was incubated with 100 mL of 9 g/L saline solution containing carboxy-methyl-cellulose (1 g/L) for 30 min at 39°C. Then SAB was detached from the plant material by using a stomacher (Stomacher 400 Circulator, Worthing, United Kingdom) for 2 min at low speed and incubated for 4 h at 4°C. Samples were centrifuged at 500 × *g* for 10 min to remove plant particles and protozoa, and the resulting supernatant was centrifuged at 10,000 × *g* for 25 min to sediment SAB. This later centrifugation was repeated after SAB resuspension. On the last day the vessel contents were sampled (50 mL) and the ammonia ^15^N enrichment was determined by the diffusion method described by Brooks et al. (1989).

### Sample Analysis

Organic matter (OM) concentration was determined by heating at 550°C for 6 h in a muffle furnace. Nitrogen and carbon concentration were measured by the Dumas combustion method (Elementar analyzer, Vario MAX cube, Hanau, Germany), while water soluble carbohydrate (WSC) concentration was measured by spectrometry using anthrone in sulfuric acid (Belanche et al., 2013a). Neutral-detergent (NDF) and acid-detergent fiber (ADF) were determined using the Automated Fiber Analyzer (ANKOM 2000, Macedon, NY, United States). Isotopic ^15^N enrichment and fermentation parameters in terms of VFA, ammonia, lactate and redox potential were determined as previously described (Belanche et al., 2016b). Oxygen pressure (log *f*) was calculated based on the redox potential (*E*_h_) and pH (Marden et al., 2013). For metabolomic fingerprinting samples were analyzed by attenuated total reflectance (ATR) from 4000 to 600 cm^-1^ using an Equinox 55 Fourier Transformed Infrared Spectrophotometer (Bruker Ltd., Coventry, United Kingdom). For the bacterial pellet samples were suspended in 200 μL distilled water; microbial pellet and cell free fraction samples were homogenized, spotted (10 μl) on to a 96 well silicone plate and dried at 40°C before scanning. For plant residues freeze dried samples were ground to a fine powder (IKA Analytical Mill, Staufer, Germany), and scanned using the Golden Gate ATR accessory (Specac Ltd., Slough, United Kingdom). Infrared settings and data collection were conducted as previously reported (Belanche et al., 2013b).

For RNA extraction, frozen samples of plant residue (for SAB) or vessel contents (for LAB) were ground to a fine powder under liquid N using a mortar and pestle before RNA was extracted using a hot phenol method (Huws et al., 2016). Then RNA (700 ng) was reverse transcribed to cDNA using random hexamer primers and Tetro Reverse Transcriptase (Bioline Ltd., London, United Kingdom) following manufacture’s guidelines. The final cDNA was diluted with 180 μL of distilled water for further analysis. Control reactions were performed without reverse transcriptase to confirm the absence of DNA in RNA samples. Absolute concentration of cDNA copies from total bacteria, anaerobic fungi, protozoa and methanogens were determined by qPCR (primers in Supplementary Table 2) as previously described (Belanche et al., 2016b). Dilutions during the RNA preparation and RT were considered for each sample in order to back calculate the absolute concentration of each microbial group per unit of original sample.

### Ion Torrent Next Generation Sequencing (NGS)

The LAB and SAB communities were studied using NGS (Belanche et al., 2016c). Amplification of the V6–V8 hypervariable region of the bacterial 16S rRNA were generated by PCR using primers 799F2 and E1163R followed by adaptors (Supplementary Table 2). Forward primers were barcoded (10 nucleotides) for sample identification. Amplification conditions for bacteria and methanogens were 95°C for 3 min, then 26 cycles of 98°C for 30 s, 58°C for 30 s and 72°C for 30 s with a final extension step of 72°C for 3 min. Library preparation and sequencing was performed using an Ion Torrent system and four PGM Sequencing 316^TM^ v2 chips (Life Technologies Ltd, Paisley, United Kingdom) as previously described (Belanche et al., 2016b). Mothur software was used to remove low quality sequences: sequences were trimmed at 400 bp, maximum of 10 homo-polymers were allowed, no mismatch with the primer/barcoding were allowed and average quality above Q15 over a 30 bp window. Error rate was controlled using UParse with the default parameter (error rate of 1). Chimera check, both *de novo* and database driven was performed using Uchime and sequences were clustered into OTUs at 97% identity using Uclust. The number of reads per sample was normalized to the sample with the lowest number of reads. Ribosomal Database Project-II (RDPII) was used to obtain the bacterial taxonomic information based on 16S data. Only annotations with a bootstrap value over 50% were assigned, otherwise they were considered as unclassified. Raw sequence reads were deposited at the EBI Short Read Archive from the European Nucleotide Archive (accession number PRJEB20255).

### Calculations and Statistical Analyses

Plant colonization by SAB and ammonia incorporation by SAB was determined based on the ^15^N enrichment ratios as follow:

SAB-N : plant N = Plant residue ^15^N enrichment : SAB ^15^N enrichment

SAB-N from NH_3_ = SAB ^15^N enrichment : NH_3_^15^N enrichment

Quantitative PCR data were log-transformed to assume normality. For the kinetic of plant colonization, feed utilization, microbial numbers and diversity, data were analyzed based on a repeated-measures procedure (REML) using Genstat 16th Edition, VSN International, United Kingdom):

$$Y_{\text{ijk}}=\mu +F_{\text{i}}+V_{\text{j}}+T_{\text{k}}+FV_{\text{ij}}+FT_{\text{ik}}+VT_{\text{jk}}+FVT_{\text{ijk}}+A_{\text{l}}+e_{\text{ijkl}}$$

where *Y*_ijk_ is the dependent, continuous variable (*n* = 4), μ is the overall mean, *F*_i_ is the fixed effect of the forage (*i* = GRA vs. HAY), *V*_j_ is the fixed effect of the vitamin E supplementation (*j* = - vs. +), *T*_k_ is the fix effect of the sampling time (*k* = 2, 4, 8, and 24 h or 48 h), *FV*_ij_, *FT*_ik_, *VT*_jk_ and *FVT*_ijk_ are their interactions, *A*_l_ is the random effect of the animal inoculum (*j* = 1, 2, 3, and 4) and *e*_ijkl_ is the residual error. When significant effects were detected across treatments, means were compared by Fisher’s protected LSD-test. Significant effects were declared at *P* < 0.05 and tendency to differences at *P* < 0.1.

Treatment effects on NGS log-transformed data were determined based on their Bray–Curtis distance metric using the UPGMA function. Data were then analyzed by non-parametric permutational multivariate analysis of variance using PRIMER-6 software (PRIMER-E Ltd., Plymouth, United Kingdom). Statistical signification was calculated after 999 random permutations of residuals under a reduced model using the Monte Carlo test. A canonical correspondence analysis (CCA) was also conducted to explore the relationships between the structure of the LAB or SAB and the fermentation pattern. The significance of each variable was also calculated using 999 random permutations (R statistics; Vegan package). For bacterial abundance distribution, data were log transformed and *P*-values were adjusted for multiple testing to decrease the False Discovery Rate (Benjamini and Hochberg, 1995).

To describe the complexity of the interaction between bacterial genera, a network analysis was performed at each time point for LAB and SAB using log-transformed data. For each time point (*n* = 8) only those genera present in more than 75% of the samples were considered. Spearman correlation analysis was performed between all bacterial genera and only correlation coefficients larger than 0.7 and adjusted *P*-values below 0.05 were further included in the correlation network. Network analysis was generated using R package igraph in which the clusters shows the community structure based on edge betweenness (measure of centrality in a graph based on shortest paths). Bacterial networks complexity was described in terms of number of nodes (genera), number of edges (positive and negative correlations), average number of neighbors and percentage that represents the sum of all genera present in the network in respect to the total community.

For metabolomics, Fourier transformed infra-red (FTIR) spectra were imported into Matlab (version 2007b, The MathWorks Inc., Natick, United States), averaged, transformed to the first Savitzky–Golay derivative to smooth baseline noise and improve spectral resolution using a 13-point window, and then mean center normalized (mean = 1, Standard Deviation = 1). Finally, differences between treatments were investigated by non-permutational multivariate analysis of variance as described before.

## Results

### Kinetics of Feed Degradation and Fermentation Pattern

**FIGURE 1:**
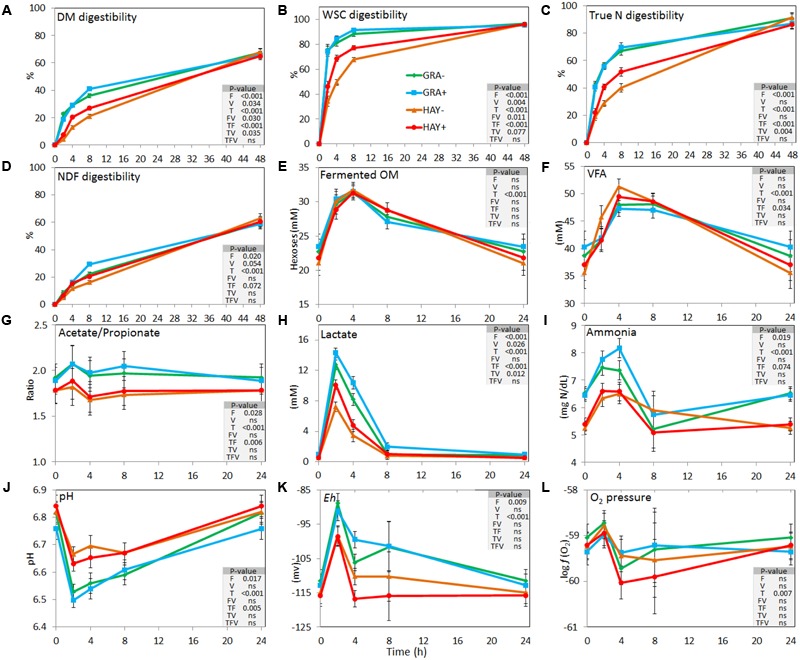
Effect of the type of forage and vitamin E supplementation on the dynamics of feed degradation **(A–D)** and fermentation pattern **(E–L)** in the Rusitec system. Error bars indicate the standard error of the mean and *P*-values are depicted for the effect of forage type (F), vitamin E supplementation (V), time points (T) and their interactions (*n* = 4).

As a result of the haymaking process HAY had lower concentrations of vitamin E (-57%) and nitrogen (-7.7%) and higher concentrations of NDF (+9.2%) and ADF (+17.1%) (Supplementary Table 1). In terms of the kinetics of feed degradability (**Figure 1** and Supplementary Table 3), GRA diets had a faster disappearance of OM, WSC, total N, true N and NDF. These differences were time-dependent and effects disappeared after 48 h of incubation (interaction F × T). Vitamin E supplementation increased disappearance of DM, WSC, total N and NDF, with these differences palpable between 4 and 8 h of incubation (V × T) and evident for HAY diets but not for GRA diets (F × V).

Concentration of fermentation products peaked between 2 and 4 h after feeding for ammonia, lactate, redox potential, fermented OM and pH, or between 4 and 8 h for VFA. Vessels fed GRA diets had higher concentrations of ammonia and lactate and higher redox potential but lower pH and propionate concentrations compared to HAY diets (**Figure 1**). A significant interaction between the type of forage and time was observed for total VFA, acetate, propionate and butyrate molar proportions, total lactate, pH and ammonia, with a higher concentration of fermentation products from GRA than for HAY diets during early fermentation (2–4 h after feeding) but no differences during the later fermentation time points (8–24 h). Vitamin E supplementation had a minor effect on the fermentation pattern and only increased total lactate concentration.

### Plant Colonization by Microbes Based on ^15^N and qPCR

**FIGURE 2:**
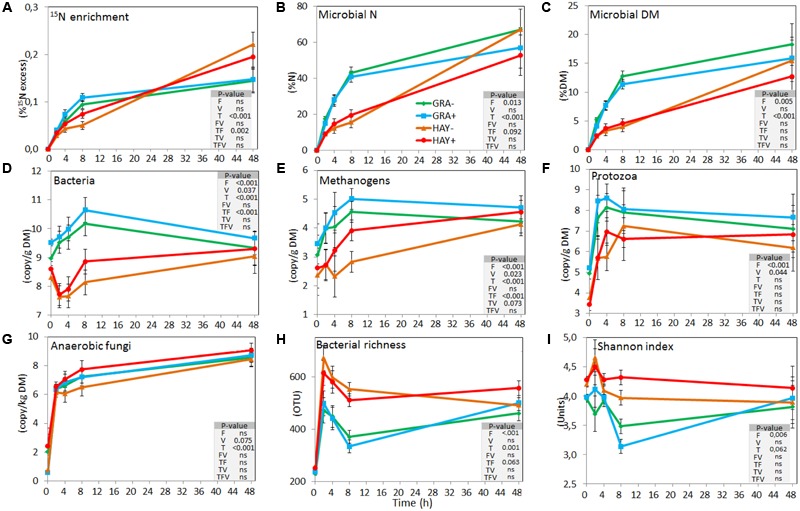
Effect of the type of forage and vitamin E supplementation on the dynamics of plant colonization by rumen microbes based on ^15^N labeling **(A–C)**, on the concentration of different microbial groups determined by qPCR **(D–G)** and on the bacterial diversity indexes based on NGS **(H,I)** in the Rusitec system. Error bars indicate the standard error of the mean and *P*-values are depicted for the effect of forage type (F), vitamin E supplementation (V), time points (T) and their interactions (*n* = 4).

Isotopic labeling of rumen microbes showed that ^15^N enrichment of plant residues progressively increased as a result of microbial colonization (**Figure 2A** and Supplementary Table 4). This was more rapid in GRA diets during the early stages and microbes represented 16, 28, and 42% of the total N in plant residue at 2, 4, and 8 h, while HAY was colonized at a slower speed (9, 14, and 17%, respectively). However, both forage types reached a similar extent of microbial N colonization after 48 h incubation (average 61%). Similar differences between forages were observed when microbial colonization was expressed in DM. The proportion of SAB-N resulting from ammonia incorporation was 17, 20, 26, and 33% for treatments GRA-, GRA+, HAY- and HAY+, respectively, thus higher for HAY than GRA diets (*P* = 0.017) with no effect of vitamin E.

**FIGURE 3:**
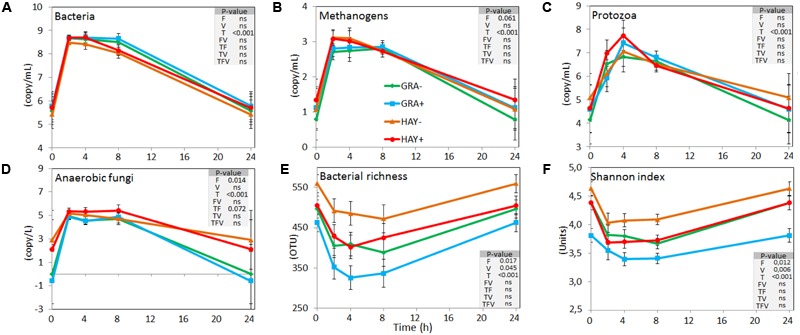
Effect of the type of forage and vitamin E supplementation on the dynamics of the liquid associated rumen microbes based on the concentration of different microbial groups determined by qPCR **(A–D)** and on the bacterial diversity indexes based on NGS **(E,F)** in the Rusitec system. Error bars indicate the standard error of the mean and *P*-values are depicted for the effect of forage type (F), vitamin E supplementation (V), time points (T) and their interactions (*n* = 4).

Quantitative PCR (**Figure 2** and Supplementary Table 4) showed a faster colonization of GRA diets by bacteria, methanogens and protozoa during the early fermentation (<8 h), while no differences compared to HAY were observed thereafter (F × T). Vitamin E supplementation increased the concentration of bacteria, methanogens, protozoa and anaerobic fungi associated with the plant residues. In the liquid phase the concentration of bacteria, methanogens, protozoa and anaerobic fungi increased after feeding (**Figure 3** and Supplementary Table 4). Although HAY diets had higher final fungal concentration in the liquid phase, the rest of the microbial groups were unaffected by the experimental treatments.

### Biodiversity and Structure of the Bacterial Communities Based on 16S cDNA Sequencing

Bacterial 16S cDNA sequencing generated 4.27 million high quality sequences after quality filtering which were distributed in a total of 1358 different OTUs. All samples were normalized at 11,265 sequences per sample. In terms of bacterial diversity (Supplementary Table 4), Chao index or Good’s coverage remained high and constant indicating that sequencing depth was comparable across all samples.

**FIGURE 4:**
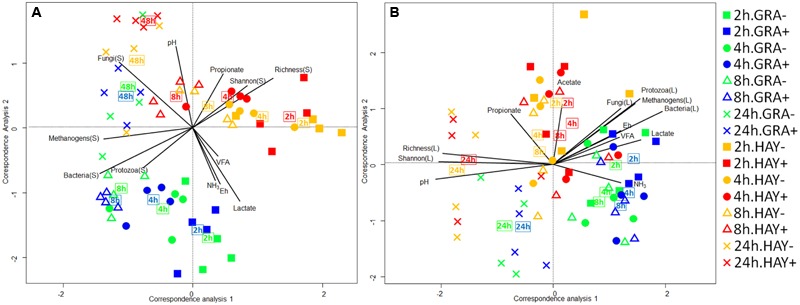
Canonical correspondence analysis illustrating the effect of grass (GRA), grass hay (HAY) and vitamin E supplementation (-,+) on the relationship of the structure of the bacterial community associated with the plant residue **(A)** or the liquid phase **(B)** with the rumen fermentation parameters in the Rusitec system. Lines show the direction and lengths are proportional to the correlations (*P* < 0.05). Centroid is indicated for each treatment.

The bacterial diversity in the epiphytic community (feed samples at 0 h) was composed of 480 and 532 OTUs in GRA and HAY diets, respectively, but once inside the vessels feeds were rapidly colonized by the rumen bacterial community which reached a maximum diversity at 2 h after feeding (**Figure 2**). HAY diets had a greater bacterial diversity indices in the SAB community in terms of richness, Shannon and Simpson index indicating the presence of a greater number of bacterial species and similar abundance of the different bacterial species (OTU’s) compared to GRA diets (Supplementary Table 4), but those differences tended to disappear at 48 h incubation (F × T). Permutational analysis of variance showed a strong effect of the F × T interaction on the structure of the SAB community (**Table 1**) indicating that differences between forage types disappeared at later stages of the colonization process (48 h). Vitamin E supplementation had no effect on the structure of the SAB community. CCA showed a clear separation of the GRA (bottom left) and HAY samples (top right) at early incubation times but progressively converged toward a similar position of the ordination plot (top left) at 48 h incubation (**Figure 4A**). Vitamin E supplementation tended to accelerate the changes in the SAB community over time as depicted by the treatments centroids. Several variables were correlated with the sample distribution in CCA: during the primary colonization (2 h after feeding) the structure of the SAB community was positively correlated with the concentration of lactate, ammonia and total VFA (*P* < 0.001). During the secondary colonization (4–8 h after feeding) SAB community was positively correlated with the concentration of bacteria, protozoa and methanogens for GRA samples but with the vessel pH, propionate molar proportion and bacterial diversity for HAY samples (*P* < 0.001). During the tertiary colonization (>8 h after feeding) SAB community was positively correlated with the concentration of anaerobic fungi (*P* < 0.001).

**Table 1:** PERMANOVA illustrating the effect of the type of forage, vitamin E supplementation and sampling time on the structure of the bacterial community associated to the plant residue and to the liquid phase and on the FTIR spectroscopy metabolic profile of the plant residue including microbes and microbial pellet and cell free fraction isolated from the liquid phase in the Rusitec system.

Analysis^1^	Bacterial 16S rRNA sequencing				FTIR spectroscopy metabolics profiling					
	Plant Residue		Liquid phase		Plant Residue		Microbial pellet		Cell free fraction	
Factors	Pseudo-F	*P*-value	Pseudo-F	*P*-value	Pseudo-F	*P*-value	Pseudo-F	*P*-value	Pseudo-F	*P*-value
Time	15.69	0.001	14.46	0.001	28.30	0.001	2.71	0.051	19.45	0.001
Forage	9.00	0.007	5.67	0.015	14.22	0.002	8.96	0.013	3.81	0.050
Vitamin E	1.23	0.292	3.03	0.041	4.94	0.006	9.19	0.003	0.56	0.675
Forage × Vitamin E	0.86	0.582	0.77	0.620	1.27	0.303	0.73	0.547	0.74	0.584
Forage × Time	2.58	0.002	1.33	0.098	1.54	0.077	2.31	0.038	7.69	0.001
Vitamin E × Time	0.90	0.627	0.96	0.528	1.04	0.402	2.04	0.083	0.76	0.658
Forage × Vitamin E × Time	0.70	0.842	0.90	0.627	0.84	0.666	0.93	0.468	1.86	0.094
Forage effect										
At 2 h	2.41	0.017	2.19	0.014	1.35	0.191	3.63	0.005	3.58	0.011
At 4 h	2.87	0.006	2.03	0.027	2.50	0.013	2.36	0.034	2.10	0.048
At 8 h	3.20	0.004	2.12	0.020	2.72	0.008	1.66	0.116	1.07	0.383
At 24 h	1.23	0.251	1.76	0.060	2.96	0.003	1.05	0.420	2.04	0.068
Vitamin E effect										
At 2 h	0.92	0.506	1.42	0.126	1.07	0.386	2.44	0.029	1.85	0.084
At 4 h	0.92	0.550	1.80	0.042	1.48	0.096	2.20	0.037	0.56	0.789
At 8 h	1.16	0.298	1.47	0.095	1.46	0.123	2.14	0.046	0.58	0.761
At 24 h	1.03	0.404	1.10	0.348	1.89	0.026	2.27	0.023	0.19	0.963

*^*1*^PERMANOVA was performed based on Bray Curtis dissimilarity. Samples were taken at 2, 4, 8, and 24 h after feeding (or 48 h for plant residue).*

In the liquid phase, LAB diversity was lowest during the fermentation peak (2–8 h after feeding, **Figure 3** and Supplementary Table 4). Similar to observed for SAB, LAB diversity was higher for HAY than GRA diets, while vitamin E supplementation decreased all diversity indices. Permanova analysis showed that the structure of LAB community was affected by time, forage type and vitamin E supplementation. Differences between both forages tended to disappear after 24 h incubation (F × V), while the effect of the vitamin E was only detected between 4 and 8 h after feeding. CCA showed a clear separation in the structure of the LAB community promoted by GRA (right) or HAY samples (left), as well as a clear shift driven by the incubation time (bottom) (**Figure 4B**). LAB community in vessels fed GRA diets was positively correlated to lactate, VFA and ammonia levels during early fermentation (2–4 h), while in HAY diets was correlated to acetate and propionate molar proportions. On the contrary, during the later fermentation time points (>24 h) LAB community was positively correlated with biodiversity indexes (*P* < 0.001) and negatively correlated to the concentration of bacteria, protozoa, methanogens and anaerobic fungi (*P* < 0.001).

### Bacterial Taxonomy

**FIGURE 5:**
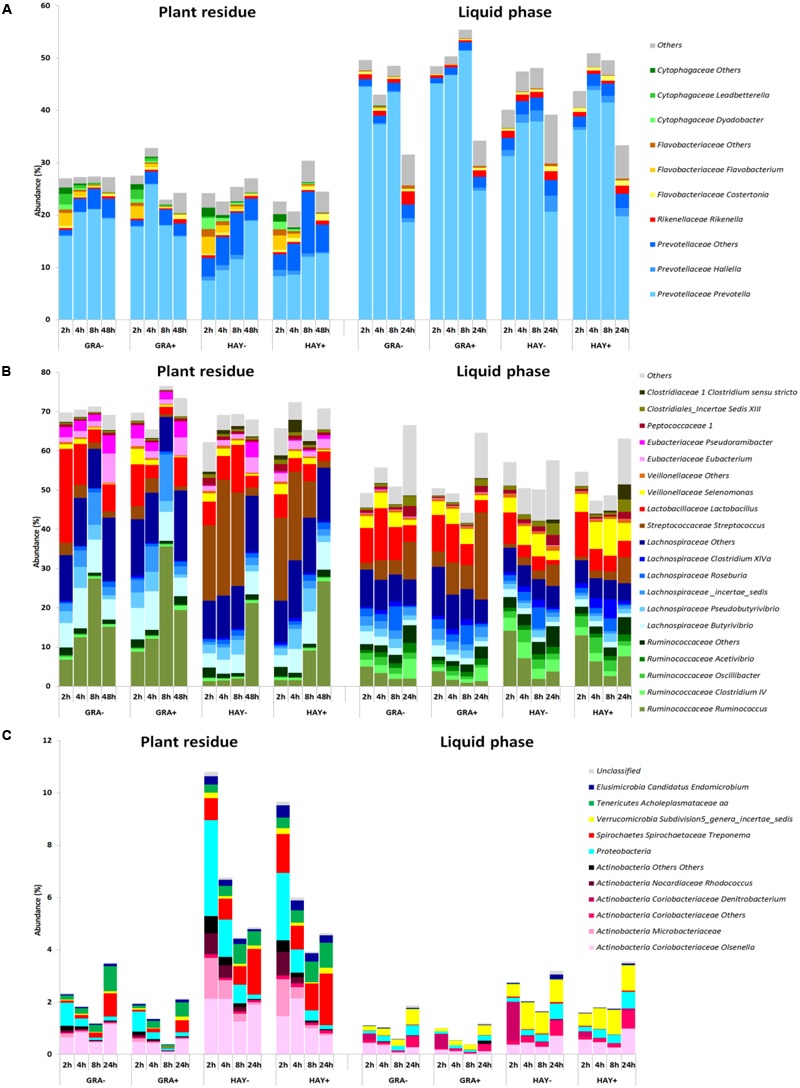
Abundance of the main bacterial genera in the phylum Bacteroidetes **(A)**, Firmicutes **(B)** and minor phyla **(C)** in the plant residue and liquid phase to describe the effect of grass (GRA), grass hay (HAY) and vitamin E supplementation (-,+) on the dynamics of plant colonization and utilization in the Rusitec system (*n* = 4).

The epiphytic bacterial community (data not shown) was dominated by Bacteroidetes (52%), Proteobacteria (21%) and Actinobacteria (12%). However, on exposure to rumen fluid this community was rapidly substituted by the SAB community which was dominated by Firmicutes (69%) and Bacteroidetes (26%). The sampling time modified the abundance of most SAB groups (**Figure 5** and Supplementary Table 5) promoting a progressive increase in the abundance of fibrolytic Ruminococcaceae while others reached a plateau at 4 h after feeding (i.e., Prevotellaceae, Lachnospiraceae) or declined over time (i.e., Proteobacteria). The type of forage used and its interaction with the incubation time promoted differences in *Lactobacillus, Streptococcus, Prevotella, Butyrivibrio, Pseudobutyrivibrio, Selenomonas*. Vitamin E supplementation had a minor effect on the general distribution and only modified the levels of few bacterial groups (i.e., *Ruminococcus*).

The LAB community was also dominated by Firmicutes (54%) and Bacteroidetes (42%) and was highly affected by the incubation time which promoted a progressive increase in Firmicutes to the detriment of Bacteroides and minor phyla (**Figure 5** and Supplementary Table 6). GRA diets increased the levels of *Prevotella, Lachnospiraceae, Butyrivibrio* and *Streptococcus* while HAY diets increased the levels of *Ruminococcaceae, Selenomonas* and *Proteobacteria*. The effect of vitamin E on the liquid associated bacteria consisted of an increase of the levels of *Prevotella* and *Clostridium XIVa* and a decrease in *Rikenella, Clostridiales, Peptococcaceae, Ruminococcaceae, Spirochaetes* and unclassified bacteria.

### Bacterial Network Analysis

**FIGURE 6:**
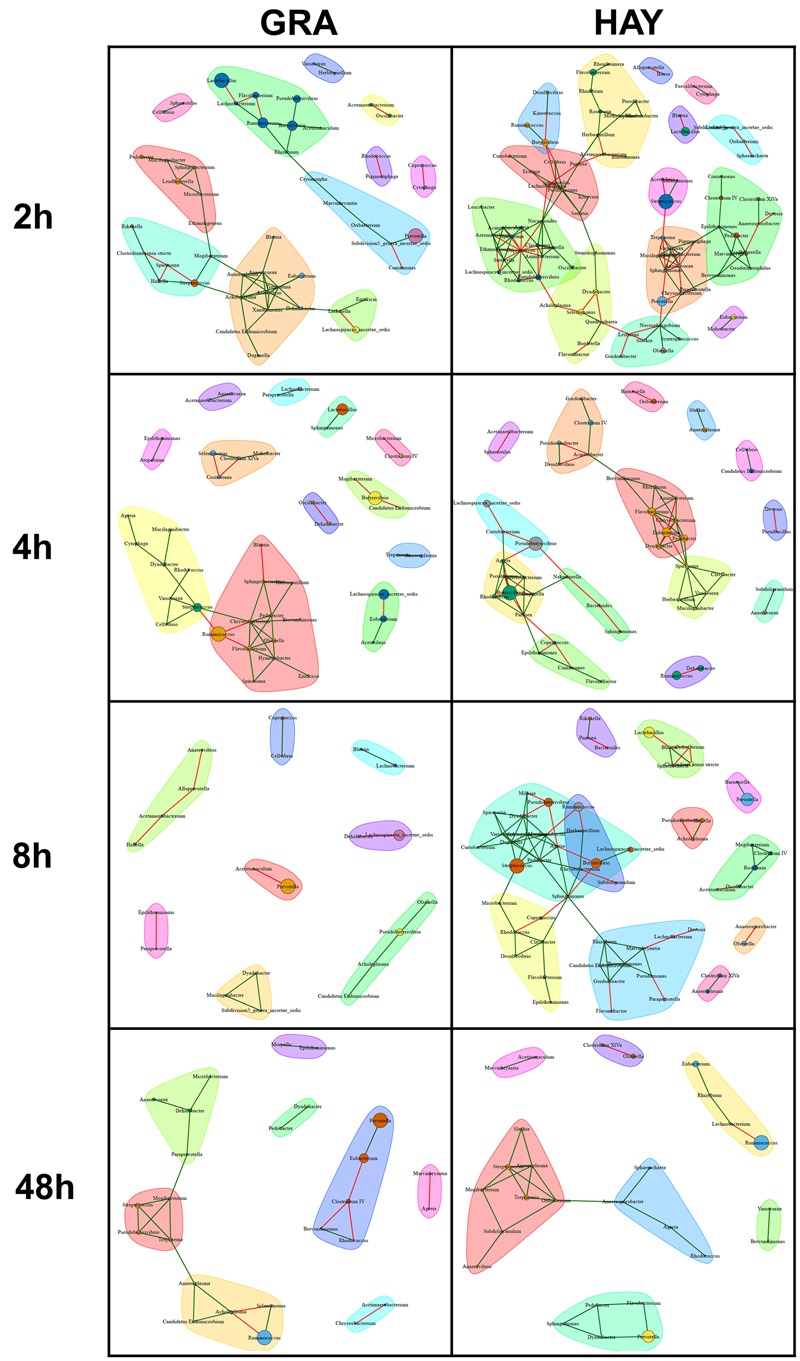
Bacterial networks illustrating the effect of grass (GRA) and grass hay (HAY) on the relationships between the different bacterial genera in the plant residue in the Rusitec system. Edges represent positive (green) and negative (red) correlation coefficients between genera (*r* > 0.7 and adjusted *P* < 0.05).

**FIGURE 7:**
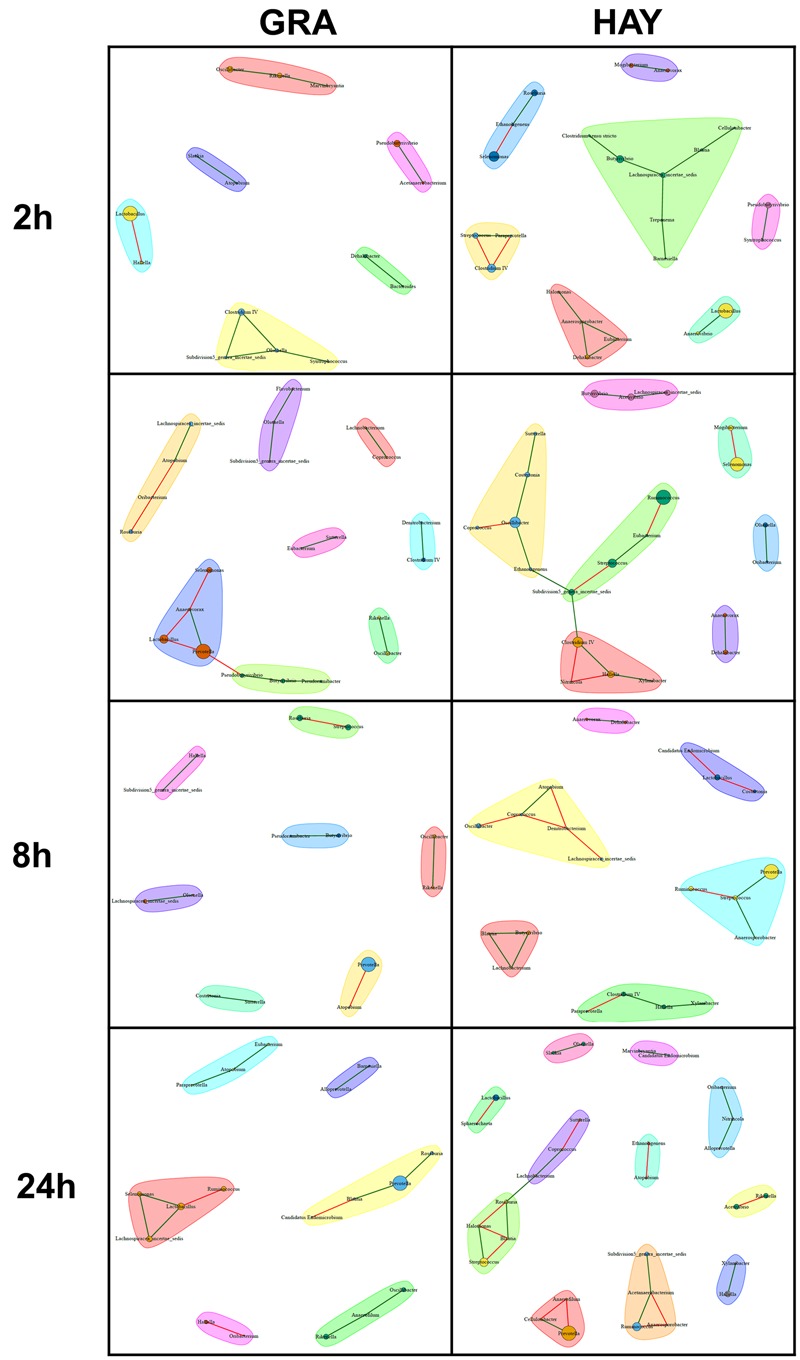
Bacterial networks illustrating the effect of grass (GRA) and grass hay (HAY) on the relationships between the different bacterial genera in the liquid phase in the Rusitec system. Edges represent positive (green) and negative (red) correlation coefficients between genera (*r* > 0.7 and adjusted *P* < 0.05).

**FIGURE 8:**
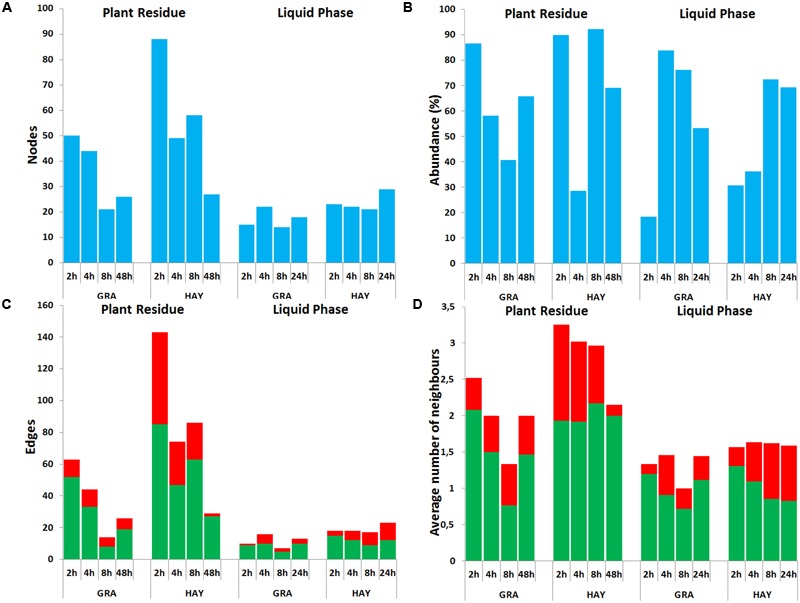
Bacterial network data describing the effect of type of forage on the dynamics of plant colonization in terms of number of nodes **(A)**, abundance **(B)**, number edges **(C)** and average number of neighbors **(D)** in the Rusitec system. Network was generated based on those genera which were positively (green) or negatively (red) correlated (*r* > 0.7 and adjusted-*P* < 0.05).

In comparison to LAB, SAB promoted a more complex bacterial network which progressively simplified over the incubation time (**Figures 6, 7**). GRA diets promoted a less complex SAB network than HAY diets at 2 h (50 vs. 88 nodes), 4 h (44 vs. 49) and 8 h (21 vs. 59) but similar values were observed at 48 h after feeding (26 vs. 27). Moreover the proportion of negative correlations tended to decrease over time in HAY (from 41 to 7%) but remained constant in GRA diets (**Figure 8**). The LAB networks (**Figures 7, 8**) maintained a simple and fairly constant composition over time. This network was more complex for HAY than for GRA diets (24 vs. 17 nodes) with these differences particularly evident 8 h after feeding. The effect of vitamin E on the SAB and LAB networks was negligible and therefore results are not presented.

### Metabolite Fingerprinting of Rumen Interactome

**FIGURE 9:**
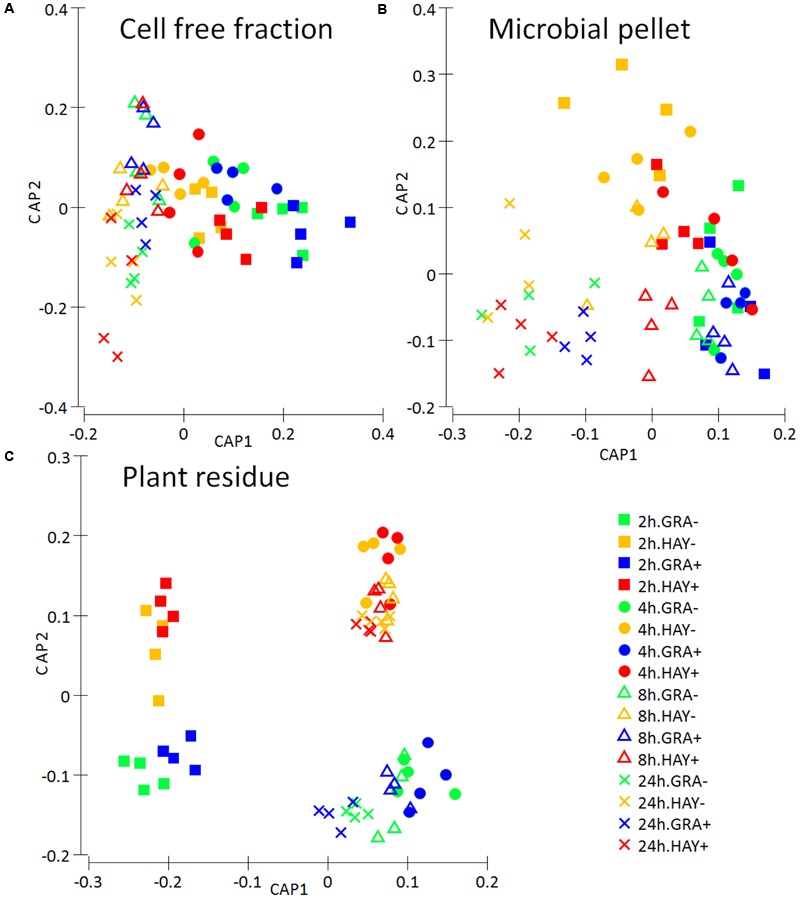
Metabolite fingerprinting based on FTIR spectroscopy depicting the effect of the type of forage and vitamin E supplementation on the dynamics the rumen interactome in the Rusitec system: a cell free fraction **(A)**, a microbial pellet recovered from the planktonic phase **(B)** and a plant residue including microorganism **(C)**. Canonical Analysis of Principal Coordinate was performed based on Bray–Curtis dissimilarity.

Analysis of the FTIR fingerprints by Permanova (**Table 1**) and CCA (**Figure 9**) clearly demonstrate that the three rumen fractions studied (plant residue, microbial pellet and cell free fraction) behaved differently in regard to discrimination between dietary treatments and time. The metabolome of plant residues was highly affected by time, with the biggest differences observed between 2 h after feeding and later time points; the type of forage, in which differences tended to increase with incubation time (F × T); and vitamin E supplementation, which promoted an acceleration in the plant colonization/degradation (**Figure 9C**). The metabolome profile in the microbial pellet followed was highly affected by the vitamin E supplementation, forage type and time. The interaction F × T showed important differences in the bacterial pellet metabolome between forage types during the early fermentation (2 and 4 h) but tended to disappear at later incubation times. Similarly an interaction F × T was observed for cell free fraction metabolome, although this fraction was mainly determined by the sampling time and forage type but unaffected by vitamin E supplementation.

## Discussion

This study, based on changes seen in both SAB and LAB communities, fermentation pattern, feed degradation and metabolomics profiles, demonstrated that forage colonization and utilization by rumen microbes is a tri-phasic process which was highly affected by the forage preservation method and could be modulated by vitamin E supplementation having important consequences for rumen function and feed utilization.

### Effects of Vitamin E on Feed Colonization and Utilization

In a previous experiment we demonstrated that vitamin E supplementation had positive effects on feed degradation after 48 h incubation in the Rusitec system (Belanche et al., 2016a). Here we have showed that vitamin E had no effect on the concentration of the major microbial groups but promoted a shift in the structure of the LAB community during the first 8 h after feeding. This shift was associated with a decrease in the bacterial diversity (-59 OTUs) suggesting that vitamin E at 50 IU/L could limit the growth of certain bacterial groups, possibly because it generates a prolonged oxidative stress to the microbes (Tagliapietra et al., 2013). Vitamin E had a negative impact on the abundance of minor bacterial species but promoted the proliferation of proteolytic bacteria such as *Prevotella* (+14%) and *Clostridium* (+83%) in the liquid phase. This effect could explain higher true N digestibility of HAY+ vs. HAY- diets during the first 8 h after feeding (+11%) but not for GRA diets due to its naturally high concentration of α-tocopherol.

Vitamin E supplementation had no effect on the structure and diversity of the SAB community but increased the concentration of total bacteria (+0.42 log), methanogens (+0.67), anaerobic fungi (+0.95) and protozoa (+0.30) in the plant residue during the first 8 h after feeding regardless of the type of forage used. Permanova and CCA also detected a slight acceleration of the plant colonization process with vitamin E supplementation, mainly during the secondary colonization (4–8 h after feeding). As a result, during this period vitamin E supplementation had the greatest positive effects in terms of DM (+19%), NDF (+25%) and true N disappearance (+13%).

### Effect of Grass vs. Hay on Primary Feed Colonization

Previous studies investigating temporal microbial colonization of air-dried switchgrass (Piao et al., 2014) and fresh grass (Edwards et al., 2007; Huws et al., 2016; Mayorga et al., 2016) described the primary colonization process within the initial 30 min and 2 h, respectively. Our study also revealed that the small epiphytic microbial community was rapidly replaced by primary colonizers; a small (less than 4% of the DM in the plant residue) but highly diverse rumen microbial community in term of rumen bacteria, methanogens, protozoa and fungi. Most of this community (88%) was part of a highly complex bacterial network. Moreover this community was particularly active as CCA showed that its population structure was positively correlated with OM fermentation and accumulation of fermentation products such as VFA, lactate and ammonia. Furthermore a similar pattern was found based on ^15^N and for total bacteria and methanogens based on qPCR suggesting that both communities share similar patterns of attachment to substrates. Firmicutes was the main phyla (69%) in the SAB community. Previous *in situ* (Piao et al., 2014; Huws et al., 2016) and *in vitro* studies (Mayorga et al., 2016) investigating changes in diversity of SAB also noted a preponderance of Firmicutes and Bacteroidetes, although Fibrobacteres abundance tended to be higher than reported here. Protozoa were the community that most rapidly colonized the diet reaching a concentration peak at 2 h for GRA diets and 4 h for HAY diets. Anaerobic fungi also rapidly colonized during the first hours after feeding. Fungi are able to directly penetrate the cuticle of plant material decreasing the tensile strength of the forage and providing additional sites for bacteria to attach to protected plant tissues during the latter stages of the colonization process (Akin and Rigsby, 1987). Soluble components diffuse from plant tissues and act as chemo-attractants, allowing fungal zoospores (Orpin and Bountiff, 1978) and protozoa (Orpin and Letcher, 1978) to locate and attach to feed particles. The ability of ciliates to freely attach and dissociate from feed particles suggests that their attachment is a purely physical phenomenon which may explain the greater abundance of protozoa associated with GRA vs. HAY residues but their similar concentration in the liquid phase.

The main event during primary feed colonization by SAB is the adhesion process. Miron et al. (2001) divided this process in four phases: (1) transport of the non-motile bacteria to the substrate; (2) initial non-specific adhesion of bacterial to unprotected sites of the substrate by elements of the glycocalyx; (3) specific adhesion to digestible tissue via extensive linkages and adhesins; (4) proliferation of the SAB on potentially digestible tissues of the substrate. Each of these phases depends on the successful completion of the previous one, thus the degree to which rumen microbes attach to and penetrate GRA and HAY physical barriers such as the silica cuticle is reflected in the fermentation lag time for each feed. Our findings based on isotopic labeling of rumen microbes revealed that primary colonization of GRA was twice as fast as HAY since at 2 h after feeding 16% of the total N in the plant residue from GRA diets was microbial N, but only 9% in HAY diets. These findings agree with qPCR data which also noted lower concentrations of total bacteria (-1.94 log), methanogens (-1.27 log) and protozoa (-2.37 log) in HAY than in GRA diets at 2 h after feeding. In terms of bacterial taxonomy, the most abundant genera during the early colonization of GRA diets were *Lactobacillus* (17.3%) and *Prevotella* (16.8%). These microbes were part of a relatively simple bacterial network (50 nodes and 63 edges) which had a higher proportion of positive correlations among bacterial genera (82% positive) perhaps as a result of high availability of easily fermentable substrates. Dipeptide degradation is considered the limiting factor in rumen proteolysis because dipeptidyl peptidase activity occurs only in very few bacterial groups (mainly, *Prevotella*) (Wallace, 1996). Thus, the higher concentration of *Prevotella* in GRA vs. HAY diets may explain greater N degradation and ammonia levels (+18%) during the primary colonization stage. Taken together, the observations suggest that primary colonizing bacteria for GRA are likely to utilize soluble nutrients and easily accessible substrates, as was confirmed by the high disappearances for WSC (75%) true N (41%) and DM (21%) but low NDF disappearance (7%).

For HAY diets the most abundant genera during the early colonization stage was *Streptococcus* (20.3%) which was part of a more complex bacterial community than observed in GRA diets in terms of diversity (+158 OTUs) and network density (+38 nodes and +80 edges) highlighting the complexity of early HAY colonization; moreover the high proportion of negative correlations (40%) suggested the importance of competition processes among bacterial groups possibly as a result of limiting availability of fermentable substrates. This observation was confirmed by the significantly lower disappearances of DM (-72%) and true N (-50%) but similar NDF disappearance for HAY in comparison to GRA diets during the primary colonization. These findings agree with most of the literature which reports lower rumen and total tract DM digestibility for cows fed hay in comparison to fresh grass (Minson, 1990; Holden et al., 1994). However, our experiment suggests that such differences mainly occurred during the first 8 h after feeding.

Fresh forage is capable of degrading part of its own protein and lipids within the first 2 h of ruminal incubation irrespective of the microbial colonization (Kingston-Smith et al., 2003; Lee et al., 2004). However, metabolome analysis of the plant residue revealed no differences between forage types at 2 h after feeding. A similar lack of differences in FTIR spectra of plant residue was observed between 2 and 4 h for most fresh ryegrass genotypes incubated *in vitro* (Kingston-Smith et al., 2013). Since FTIR spectra is highly correlated with the chemical composition (Belanche et al., 2014), those differences in the primary colonization seems to be mainly triggered by the physical plant damage and accessibility of the microbes to the plant rather than to the chemical composition “*per se*”.

### Effect of Grass vs. Hay on Secondary Feed Colonization

Several authors have described secondary colonization as events occurring between 30 min and 4 h *in sacco* (Piao et al., 2014) and between 2 and 4 h after feeding *in vitro* (Mayorga et al., 2016). Our *in vitro* findings suggested that between the 2 and 4 h after feeding interval a series of events occurred. This secondary colonization was a dynamic and linear process in which the SAB community increased in size but decreased in diversity (-122 OTUs) indicating a predominance of fewer bacterial groups which took part in a progressively simpler bacterial network composed of half of the bacterial community. Despite these general features, important differences between GRA and HAY diets were noted.

Isotopic labeling of microbial N showed a 3× slower rate of secondary colonization with HAY diets as during this interval the contribution of microbial N in the plant residue increased by 26% for GRA diets but only 8.4% for HAY diets. Moreover, the structure of the bacterial community differed between the forage types: according to CCA the bacterial community in GRA diets was positively correlated with increased levels of bacteria (+1.91 log), methanogens (+1.42) and protozoa (+1.06) indicating a more effective colonization process than in HAY diets. Previous studies using fresh grass have reported increased numbers of certain bacterial groups (*Ruminococcus, Pseudobutyrivibrio* and *Roseburia*) during the secondary colonization *in sacco* (Huws et al., 2016) and *in vitro* (*Prevotella, Butyrivibrio, Blautia, Bacillus* and *Acidaminococcus*) which have been associated with expression of genes involved in amino acid, carbohydrate and lipid storage (Mayorga et al., 2016). Our study generally agrees with these patterns suggesting that secondary colonization is a transition phase in which the primary colonizers described above were progressively replaced by the secondary colonizers such as *Pseudobutyrivibrio* (+60%) and *Butyrivibrio* (+40%) which dominated the bacterial network across diets. *Pseudobutyrivibrio* possess xylanases which randomly cleave the β-1,4 backbone of the complex pant cell wall polysaccharide xylan (Krause et al., 2003), thus it might favor feed degradation during secondary colonization. Moreover, during this interval the HAY diet promoted a more complex bacterial community (+179 OTUs) and network than the GRA diet in terms of nodes (+21), edges (+51) and a predominance of positive correlations (67%) among the bacterial groups indicating a greater complexity of its secondary colonization. Since secondary colonizers are likely to be adept at degrading plant structural components, this increase in diversity and network complexity in HAY diets may compensate for the delay in the colonization process in terms of microbial numbers leading to similar disappearance of DM (18%) and true N (26%) during the 4–8 h interval for both forages. It has been suggested that once the primary colonization has occurred, a more general pattern of plant tissues digestion by rumen bacteria occurs as follow: mesophyll, phloem > epidermis, parenchymal bundle sheath > sclerenchyma > lignified vascular xylem (Akin, 1989). Thus the secondary colonization may be affected by accessibility of those plant structures across treatments.

### Effect of Grass vs. Hay on Tertiary Feed Colonization

In this study the concept of tertiary colonization has been introduced to describe events which occurred later than 8 h after feeding consisting of a progressive slowdown in the colonization process and simplification of the SAB network. Sun et al. (2008) using cannulated goats also noted a marked change in the rumen bacterial community 12 h post-feeding. Our findings indicated that the structure of the SAB community during this stage was positively correlated with the concentration of anaerobic fungi, which due to their longer life cycle and ability to digest recalcitrant lignocellulosic substrates are efficient feed degraders during late colonization (Edwards et al., 2008). This SAB community was also correlated with a higher pH and thus favored the proliferation of Firmicutes (67%) such as *Ruminococcus* and *Butyrivibrio*. These microbes were part of a much simpler bacterial network (27 nodes and 27 edges) than described for earlier incubation times reflecting the scarcity of fermentable substrates at 48 h after feeding. *Ruminococcus* is well recognized for its fibrolytic capacity due to the possession of numerous glycosyl hydrolases (Dai et al., 2015) while *Butyrivibrio* is known to have proteolytic and hemicellulolytic activity (Krause et al., 2003). These two fibrolytic bacteria represented a greater proportion of the total population in HAY than GRA diets (30% vs. 20%). Indeed, isotopic labeling of microbial N revealed a 2× faster colonization of HAY vs. GRA diets during the 8–48 h interval (42% vs. 20%) as well as a higher level of total bacteria (+1.58 log) and methanogens (+1.29 log). The increased levels of *Prevotella* during the tertiary colonization of HAY diets may indicate a greater accessibility to the slowly degradable carbohydrates and proteins and acceleration in its degradation as a result of the proteolytic and glycosylic activity of this bacterium. This acceleration meant that HAY diets reached similar degrees of colonization to GRA diets at 48 h after feeding (61% of the total N). As a result, HAY diets showed higher disappearances for DM (+52%), true N (+108%) and NDF (+29%) during the 8–48 h interval. A similar acceleration in DM degradation has been reported for air-dried switchgrass hay after 16 h incubation *in sacco* (Piao et al., 2014). Moreover, Hess et al. (2011) using a similar substrate incubated for 72 h noted that 57% of the carbohydrate-active enzymes were putative celluloses highlighting the importance of the fibrolytic process during the tertiary colonization of hay. Our metabolomic analysis of plant residue revealed a progressive increase in the separation of GRA and HAY samples as the incubation time progressed possibly because of changes in the chemical composition of the plant residue and/or SAB triggered by differential colonization processes. We are aware that our *in vitro* experiment may overestimate the length of the colonization process due to the lower microbial density and absence of mastication and plant tissue damage in comparison to *in vivo* studies. However, similarity in the plant colonization events *in vivo* and *in vitro* has been reported (Piao et al., 2014; Huws et al., 2016; Mayorga et al., 2016), thus our findings must be further considered for developing strategies to optimize feed degradation efficiency.

### Effect of Grass vs. Hay on the Liquid Phase

Early DNA-based reported lower diversity for LAB than SAB community in sheep fed orchard hay and concentrate (Larue et al., 2005). Our study based on RNA sequencing confirmed this observation (-72 OTUs) as well as uncovering a much simpler bacterial network for LAB than SAB which may indicate a more limited interaction between the different bacterial groups as a result of a greater dilution and ultimately a lower physical contact among them. Despite this, the LAB community is the first microbial community which interacts with newly ingested forage. Thus, the initial stage of feed colonization is dependent on the LAB community, and more specifically on the concentration of viable bacterial cells containing a pre-adapted attachment mechanism (i.e., specific glycocalyx organelles and adhesins) which can accelerate the colonization process (Miron et al., 2001).

Our experiment demonstrated that LAB population dynamics could be described as a biphasic process. An initial phase from 2 to 8 h after feeding represented the early-mid fermentation in which the concentration of bacteria, methanogens, protozoa and anaerobic fungi reached the highest levels whilst bacterial diversity was at its lowest. Bacteroidetes, and more specifically *Prevotella* with 41% of the reads dominated the bacterial community and their levels peaked in synchrony with the ammonia levels confirming its importance in the protein metabolism in the liquid phase of the rumen. This LAB community was particularly active in GRA diets since CCA showed a positive correlation with the concentration of fermentation products such as VFA, ammonia and lactate. The GRA diet promoted a more acidic fermentation characterized by an extended period with pH below 6.5 and lactate levels above 5 mM in comparison to HAY diets (approximately 8 h vs. 2 h), possibly as result of higher levels of lactic acid producers such as *Lactobacillus* (+50%) and *Streptococcus* (+167%) but lower levels of lactic acid consumers such as *Selenomonas* (-45%). On the contrary the LAB community with HAY diets was correlated with acetate production indicating utilization of structural carbohydrates as the primary substrate (Noziere et al., 2011). Moreover HAY diets promoted a higher concentration of anaerobic fungi and methanogens which is in line with the higher fiber digestibility and methane emissions previously reported (Belanche et al., 2016a).

The second phase (>8 h after feeding) represented the late fermentation in which the bacterial community reached its highest diversity. This community had increased levels of Firmicutes indicating a transfer from the SAB community as a result of the release of feed particles rich in microbes, key for starting the next colonization cycle of new plant material (Minson, 1990). Although the LAB network was very simple across diets, it varied over time and peaked at 2–4 h for GRA diets and at 8–24 h for HAY diets representing over 70% of the entire bacterial community suggesting that the most abundant species (i.e., *Prevotella, Lactobacillus* and *Streptococcus*) were actively involved in the feed utilization. Moreover, a progressive increase in the proportion of negative correlation over time was noted for HAY diets but not for GRA diets suggesting a progressive scarcity of substrates (i.e., proteins and amino acids) in liquid phase which may explain the greater utilization of ammonia by microbes as N source for HAY vs. GRA diets (+49%).

## Implications

This study demonstrated that a holistic approach based on a detailed characterization of the temporal dynamics of the rumen microbiome coupled with an integral description of the kinetics of feed degradation and fermentation pattern are key to understand the mode of action of nutritional strategies to optimize feed utilization. It was observed that liquid- and solid-associated bacteria play a different but complementary role in forage utilization. This study noted that the forage colonization by microbes followed a tri-phasic process which was affected by the forage conservation method (grass vs. hay). Grass promoted a more rapid primary colonization and efficient nutrient utilization during the first 2 h after feeding, secondary colonization was a transition phase (4–8 h), and hay promoted an acceleration during the tertiary colonization (>8 h). Vitamin E supplementation slightly accelerated the colonization pattern during the secondary colonization leading to a small increase in feed degradability. Although these findings should be confirmed *in vivo*, our results suggest that grass should be preferentially used instead of hay, in order to accelerate feed utilization by rumen microbes; alternatively new strategies should be developed to improve the physical-chemical structure of conserved forages and/or the cellulolytic capacity of rumen microbiota.

## Author Contributions

AB, AK-S, and CN designed the experiment. AB conducted the research and wrote the manuscript. AB, WL, and PRS conducted the analyses. AB, AK-S, and CN reviewed the manuscript. AB had primary responsibility for the final content. All authors read and approved the final manuscript.

## Conflict of Interest Statement

The authors declare that the research was conducted in the absence of any commercial or financial relationships that could be construed as a potential conflict of interest.
