# Supplementary material for: A Systems Biology Approach Reveals Differences in the Dynamics of Colonization and Degradation of Grass vs. Hay by Rumen Microbes with Minor Effects of Vitamin E Supplementation
Source: Front Microbiol. 2017 Aug 3;8:1456. doi: 10.3389/fmicb.2017.01456 (PMC5541034; doi:10.3389/fmicb.2017.01456)
Supplement: Supplementary file 1 [file Table_1.DOCX]

**SUPPLEMENTAL MATERIAL**

**Supplementary Table 1**. Chemical composition of the experimental diets (in % of DM unless stated).

|  | **Grass** | **Grass hay** | **Concentrate^1^** |
| --- | --- | --- | --- |
| Dry matter (% FM) | 17.5 | 84.9 | 88.6 |
| Organic matter | 91.2 | 91.0 | 93.8 |
| Nitrogen | 1.83 | 1.69 | 2.51 |
| Carbon | 43.8 | 43.5 | 44.7 |
| Neutral detergent fibre | 50.0 | 54.6 | 39.3 |
| Acid detergent fibre | 24.0 | 28.1 | 12.1 |
| Vitamin E (IU/kg DM) | 72.3 | 30.8 | ND |

^1^Commercial concentrate made of: wheat 44.25, barley 15, palm kernel expeller 14, rapeseed expeller 11.7, maize meal 7.5, wheat-feed 5, limestone flour 1.95, NaHCO_3_ 0.3, NaCl 0.15 and NH_4_Cl 0.15% in DM. ND, not detected (Celtic Pride Premium Beef Nuts, UK).

**Supplementary Table 2**. Primers used for quantitative PCR and Ion-Torrent Next Generation Sequencing.

| **Target** | **Author** | **Forward Primer** | **Reverse Primer** | **T^a^** | | **Amplicon (bp)** |
| --- | --- | --- | --- | --- | --- | --- |
| **Quantitative PCR** |  |  |  | |  |  |
| Bacteria | ([Maeda *et al.*, 2003](#_ENREF_5)) | GTGSTGCAYGGYTGTCGTCA | ACGTCRTCCMCACCTTCCTC | | 61 | 150 |
| Methanogens | ([Denman *et al.*, 2007](#_ENREF_3)) | TTCGGTGGATCDCARAGRGC | GBARGTCGWAWCCGTAGAATCC | | 56 | 140 |
| Protozoa | ([Sylvester *et al.*, 2004](#_ENREF_6)) | GCTTTCGWTGGTAGTGTATT | CTTGCCCTCYAATCGTWCT | | 55 | 223 |
| Anaerobic fungi | ([Denman & McSweeney, 2006](#_ENREF_2)) | GAGGAAGTAAAAGTCGTAACAAGGTTTC | CAAATTCACAAAGGGTAGGATGATT | | 62 | 120 |
| **Ion Torrent NGS** |  |  |  | |  |  |
| Bacterial primers | ([Edwards *et al.*, 2007](#_ENREF_4), [Bayer *et al.*, 2014](#_ENREF_1)) | AACAGGATTAGATACCCTG | CGTCRTCCCCRCCTTCC | | 58 | 456 |
| Bacterial adaptors |  | CCATCTCATCCCTGCGTGTCTCCGACTCAG | CCTCTCTATGGGCAGTCGGTGAT | |  |  |

**Supplementary Table 3.** Effect of the type of forage and vitamin E supplementation on the dynamics of feed degradability (in % of DM) the Rusitec system

| **Treatment** | **GRA-** | | | | **GRA+** | | | | **HAY-** | | | | **HAY+** | | | |  | **P-value** | | | | | | |
| --- | --- | --- | --- | --- | --- | --- | --- | --- | --- | --- | --- | --- | --- | --- | --- | --- | --- | --- | --- | --- | --- | --- | --- | --- |
| **Time** | **2 h** | **4 h** | **8 h** | **24 h** | **2 h** | **4 h** | **8 h** | **24 h** | **2 h** | **4 h** | **8 h** | **24 h** | **2 h** | **4 h** | **8 h** | **24 h** | **SED^1^** | **F** | **V** | **F×V** | **T** | **T×F** | **T×V** | **T×F×V** |
| **Dissapearence (%)** |  |  |  |  |  |  |  |  |  |  |  |  |  |  |  |  |  |  |  |  |  |  |  |  |
| DM | 22.8 | 28.8 | 36.1 | 67.3 | 18.9 | 29.0 | 41.1 | 65.7 | 4.2 | 12.9 | 21.0 | 67.8 | 7.5 | 20.3 | 27.0 | 64.9 | 2.46 | <0.001 | 0.034 | 0.03 | <0.001 | <0.001 | 0.035 | 0.232 |
| WSC | 75.8 | 81.0 | 88.3 | 96.5 | 74.3 | 84.3 | 91.3 | 95.2 | 34.3 | 49.4 | 67.8 | 96.1 | 46.1 | 68.6 | 77.1 | 95.9 | 4.01 | <0.001 | 0.004 | 0.011 | <0.001 | <0.001 | 0.077 | 0.264 |
| Total N | 23.5 | 32.5 | 41.1 | 71.4 | 22.6 | 32.0 | 45.2 | 70.3 | 8.4 | 18.6 | 28.4 | 74.0 | 14.4 | 28.1 | 35.9 | 71.8 | 2.02 | <0.001 | 0.003 | 0.008 | <0.001 | <0.001 | 0.016 | 0.089 |
| True N | 42.0 | 56.7 | 66.9 | 90.9 | 40.7 | 55.7 | 69.7 | 86.9 | 19.1 | 28.9 | 40.0 | 91.4 | 22.1 | 40.9 | 51.8 | 86.2 | 2.89 | <0.001 | 0.350 | 0.207 | <0.001 | <0.001 | 0.004 | 0.123 |
| NDF | 9.00 | 14.6 | 22.2 | 59.7 | 5.64 | 16.3 | 29.4 | 58.8 | 5.29 | 11.61 | 16.3 | 62.7 | 6.90 | 15.6 | 20.6 | 60.6 | 2.89 | <0.001 | 0.350 | 0.207 | <0.001 | <0.001 | 0.004 | 0.123 |
| ADF | 5.82 | 8.73 | 13.8 | 53.8 | 2.11 | 9.21 | 21.6 | 52.2 | 3.99 | 5.30 | 7.65 | 57.8 | 3.51 | 8.69 | 11.8 | 54.4 | 4.07 | 0.092 | 0.400 | 0.908 | <0.001 | 0.12 | 0.188 | 0.634 |
| **Fermentation pattern** |  |  |  |  |  |  |  |  |  |  |  |  |  |  |  |  |  |  |  |  |  |  |  |  |
| pH | 6.53 | 6.56 | 6.59 | 6.82 | 6.50 | 6.54 | 6.61 | 6.76 | 6.67 | 6.70 | 6.67 | 6.82 | 6.63 | 6.65 | 6.67 | 6.84 | 0.023 | 0.017 | 0.584 | 0.9 | <0.001 | 0.005 | 0.272 | 0.154 |
| Eh (mV) | -88.8 | -106.2 | -101.6 | -111.5 | -91.1 | -99.5 | -101.3 | -112.8 | -98.3 | -110.3 | -110.3 | -114.8 | -98.6 | -116.8 | -115.8 | -115.8 | 5.48 | 0.009 | 0.641 | 0.44 | <0.001 | 0.377 | 0.863 | 0.432 |
| O_2_ pressure (Log f) | -58.2 | -59.2 | -58.8 | -58.5 | -58.5 | -58.9 | -58.7 | -58.9 | -58.3 | -58.9 | -59.0 | -58.7 | -58.4 | -59.5 | -59.4 | -58.7 | 0.34 | 0.116 | 0.151 | 0.262 | 0.007 | 0.445 | 0.934 | 0.264 |
| Ammonia N (mg/dL) | 7.47 | 7.36 | 5.22 | 6.53 | 7.76 | 8.17 | 5.75 | 6.46 | 6.34 | 6.49 | 5.90 | 5.26 | 6.60 | 6.59 | 5.09 | 5.39 | 0.473 | 0.019 | 0.625 | 0.459 | <0.001 | 0.074 | 0.514 | 0.346 |
| VFA (mM) | 41.6 | 48.0 | 48.1 | 38.7 | 41.9 | 47.3 | 47.0 | 40.3 | 45.8 | 51.3 | 48.6 | 35.6 | 41.5 | 49.5 | 48.6 | 37.0 | 1.98 | 0.731 | 0.751 | 0.741 | <0.001 | 0.034 | 0.335 | 0.503 |
| Acetate (%) | 42.6 | 41.4 | 40.9 | 41.2 | 43.6 | 42.6 | 42.8 | 41.8 | 44.2 | 42.2 | 42.5 | 43.2 | 44.2 | 42.2 | 43.1 | 43.3 | 0.44 | 0.134 | 0.292 | 0.468 | <0.001 | 0.032 | 0.224 | 0.69 |
| Propionate (%) | 20.8 | 21.6 | 21.1 | 21.7 | 21.2 | 21.7 | 21.0 | 22.2 | 24.3 | 25.1 | 24.5 | 24.2 | 23.5 | 24.7 | 24.4 | 24.4 | 0.32 | 0.02 | 0.974 | 0.81 | 0.002 | 0.043 | 0.282 | 0.357 |
| Butyrate (%) | 19.5 | 20.6 | 20.9 | 21.0 | 18.1 | 18.5 | 18.7 | 19.5 | 19.1 | 20.4 | 20.1 | 19.7 | 19.0 | 20.4 | 19.9 | 19.4 | 0.34 | 0.85 | 0.233 | 0.288 | <0.001 | 0.002 | 0.539 | 0.476 |
| Iso-butyrate (%) | 0.96 | 0.85 | 0.84 | 0.95 | 0.95 | 0.84 | 0.85 | 0.90 | 0.98 | 0.91 | 0.90 | 0.95 | 0.96 | 0.92 | 0.91 | 0.93 | 0.020 | 0.05 | 0.567 | 0.77 | <0.001 | 0.019 | 0.134 | 0.584 |
| Iso-valerate | 3.31 | 3.10 | 3.28 | 3.66 | 3.15 | 3.03 | 3.22 | 3.41 | 3.35 | 3.14 | 3.18 | 3.63 | 3.59 | 3.38 | 3.44 | 3.73 | 0.095 | 0.248 | 0.79 | 0.213 | <0.001 | 0.297 | 0.256 | 0.944 |
| Valerate (%) | 4.63 | 4.96 | 4.45 | 4.62 | 4.50 | 4.91 | 4.54 | 4.52 | 4.63 | 4.94 | 4.85 | 4.65 | 4.99 | 5.49 | 4.96 | 5.01 | 0.128 | 0.542 | 0.514 | 0.519 | <0.001 | 0.292 | 0.303 | 0.466 |
| Caproate (%) | 5.43 | 5.24 | 5.33 | 3.73 | 5.20 | 5.30 | 5.26 | 3.74 | 2.56 | 2.55 | 2.65 | 2.04 | 2.79 | 2.54 | 2.37 | 1.82 | 0.236 | 0.002 | 0.913 | 0.995 | <0.001 | 0.004 | 0.718 | 0.397 |
| Heptanoate (%) | 2.68 | 2.72 | 2.98 | 2.77 | 2.85 | 3.14 | 3.29 | 3.03 | 1.15 | 1.21 | 1.37 | 1.39 | 1.40 | 1.29 | 1.28 | 1.37 | 0.192 | 0.006 | 0.71 | 0.798 | 0.209 | 0.307 | 0.674 | 0.478 |
| Total Lactate (mM) | 12.7 | 8.20 | 0.98 | 0.87 | 14.3 | 10.4 | 1.97 | 0.98 | 7.24 | 3.46 | 0.78 | 0.56 | 10.1 | 4.79 | 1.00 | 0.54 | 0.636 | <0.001 | 0.026 | 0.896 | <0.001 | <0.001 | 0.012 | 0.309 |
| D-Lactate (mM) | 4.85 | 3.65 | 0.40 | 0.41 | 5.88 | 5.04 | 0.98 | 0.42 | 2.15 | 1.22 | 0.32 | 0.24 | 3.06 | 1.54 | 0.39 | 0.17 | 0.410 | <0.001 | 0.076 | 0.427 | <0.001 | <0.001 | 0.093 | 0.555 |
| L Lactate (mM) | 7.89 | 4.55 | 0.57 | 0.46 | 8.45 | 5.36 | 0.99 | 0.56 | 5.09 | 2.24 | 0.46 | 0.32 | 7.07 | 3.25 | 0.61 | 0.36 | 0.456 | 0.001 | 0.036 | 0.545 | <0.001 | <0.001 | 0.085 | 0.273 |

^1^Standard error of the difference for the interaction between the type of forage (F). vitamin E supplementation at 50 IU/d (V) and incubation time (T) (n=4). Feed disappearance was determined at 2. 4. 8 and 48 h after feeding.

**Supplementary Table 4.** Effect of the type of forage and vitamin E supplementation on the kinetics of plant colonization in terms of abundance of rumen microbes in the plant residue and liquid phase in the Rusitec system

| **Treatment** | **GRA-** | | | | **GRA+** | | | | **HAY-** | | | | **HAY+** | | | |  | **P-value** | | | | | | |
| --- | --- | --- | --- | --- | --- | --- | --- | --- | --- | --- | --- | --- | --- | --- | --- | --- | --- | --- | --- | --- | --- | --- | --- | --- |
| **Time** | **2 h** | **4 h** | **8 h** | **24 h** | **2 h** | **4 h** | **8 h** | **24 h** | **2 h** | **4 h** | **8 h** | **24 h** | **2 h** | **4 h** | **8 h** | **24 h** | **SED^1^** | **F** | **V** | **F×V** | **T** | **T×F** | **T×V** | **T×F×V** |
| **PLANT RESIDUE** |  |  |  |  |  |  |  |  |  |  |  |  |  |  |  |  |  |  |  |  |  |  |  |  |
| Enrichment (%^15^N excess) | 0.04 | 0.06 | 0.09 | 0.14 | 0.04 | 0.07 | 0.11 | 0.15 | 0.03 | 0.04 | 0.05 | 0.22 | 0.03 | 0.05 | 0.07 | 0.20 | 0.018 | 0.925 | 0.514 | 0.781 | <0.001 | 0.002 | 0.314 | 0.549 |
| Microbial N colonization (%) | 17.6 | 27.3 | 43.0 | 66.9 | 15.0 | 28.1 | 40.8 | 56.8 | 8.99 | 12.3 | 15.6 | 67.0 | 9.22 | 14.7 | 19.4 | 52.7 | 7.660 | 0.013 | 0.497 | 0.844 | <0.001 | 0.092 | 0.262 | 0.734 |
| Microbial DM colonization (%) | 5.24 | 7.60 | 12.8 | 18.3 | 4.10 | 7.69 | 11.3 | 15.9 | 2.39 | 3.26 | 3.99 | 15.4 | 2.42 | 3.71 | 4.57 | 12.7 | 2.452 | 0.005 | 0.502 | 0.741 | <0.001 | 0.181 | 0.515 | 0.798 |
| Bacteria (copy/g DM) | 9.52 | 9.70 | 10.2 | 9.33 | 9.70 | 9.98 | 10.6 | 9.67 | 7.63 | 7.66 | 8.14 | 9.03 | 7.72 | 7.91 | 8.86 | 9.30 | 0.286 | <0.001 | 0.037 | 0.981 | <0.001 | <0.001 | 0.421 | 0.885 |
| Methanogens (copy/g DM) | 3.99 | 4.03 | 4.57 | 4.22 | 4.00 | 4.53 | 5.01 | 4.71 | 2.74 | 2.32 | 2.83 | 4.12 | 2.71 | 3.22 | 3.91 | 4.56 | 0.302 | <0.001 | 0.023 | 0.519 | <0.001 | <0.001 | 0.073 | 0.574 |
| Protozoa (copy/g DM) | 7.66 | 8.14 | 7.91 | 7.10 | 8.46 | 8.61 | 8.06 | 7.66 | 5.69 | 5.75 | 7.24 | 6.19 | 5.70 | 6.97 | 6.61 | 6.83 | 0.716 | <0.001 | 0.044 | 0.921 | 0.320 | 0.223 | 0.519 | 0.505 |
| Anaerobic fungi (copy/kg DM) | 3.39 | 3.60 | 4.24 | 5.57 | 3.32 | 3.83 | 4.22 | 5.73 | 3.15 | 3.06 | 3.50 | 5.43 | 3.54 | 4.05 | 4.74 | 6.07 | 0.210 | 0.832 | 0.075 | 0.126 | <0.001 | 0.571 | 0.147 | 0.222 |
| **Bacterial diversity** |  |  |  |  |  |  |  |  |  |  |  |  |  |  |  |  |  |  |  |  |  |  |  |  |
| Richness | 472 | 498 | 671 | 614 | 448 | 441 | 598 | 580 | 371 | 334 | 552 | 511 | 460 | 500 | 492 | 556 | 48.72 | <0.001 | 0.843 | 0.635 | 0.001 | 0.063 | 0.294 | 0.654 |
| Shannon | 3.69 | 4.11 | 4.65 | 4.50 | 3.92 | 3.98 | 4.09 | 4.28 | 3.48 | 3.14 | 3.97 | 4.32 | 3.82 | 3.96 | 3.89 | 4.14 | 0.347 | 0.006 | 0.408 | 0.749 | 0.062 | 0.169 | 0.908 | 0.347 |
| Evenness | 0.60 | 0.66 | 0.72 | 0.70 | 0.64 | 0.65 | 0.64 | 0.67 | 0.59 | 0.54 | 0.63 | 0.69 | 0.62 | 0.64 | 0.63 | 0.65 | 0.050 | 0.034 | 0.341 | 0.680 | 0.175 | 0.219 | 0.957 | 0.310 |
| Simpson | 0.86 | 0.95 | 0.94 | 0.94 | 0.94 | 0.95 | 0.90 | 0.93 | 0.89 | 0.85 | 0.92 | 0.96 | 0.92 | 0.91 | 0.90 | 0.89 | 0.051 | 0.443 | 0.423 | 0.981 | 0.601 | 0.203 | 0.660 | 0.332 |
| Chao | 657 | 844 | 854 | 797 | 690 | 802 | 828 | 783 | 515 | 522 | 867 | 763 | 603 | 771 | 767 | 703 | 122.2 | 0.060 | 0.655 | 0.128 | 0.196 | 0.172 | 0.719 | 0.884 |
| Good's | 0.64 | 0.61 | 0.71 | 0.72 | 0.62 | 0.61 | 0.67 | 0.68 | 0.64 | 0.61 | 0.62 | 0.66 | 0.68 | 0.65 | 0.64 | 0.71 | 0.043 | 0.043 | 0.845 | 0.132 | 0.189 | 0.186 | 0.842 | 0.747 |
| **LIQUID PHASE** |  |  |  |  |  |  |  |  |  |  |  |  |  |  |  |  |  |  |  |  |  |  |  |  |
| Bacteria (copy/mL) | 8.67 | 8.61 | 8.50 | 5.58 | 8.68 | 8.70 | 8.65 | 5.79 | 8.47 | 8.41 | 8.05 | 5.43 | 8.68 | 8.70 | 8.15 | 5.70 | 0.233 | 0.141 | 0.203 | 0.677 | <0.001 | 0.292 | 0.794 | 0.797 |
| Methanogens (copy/mL) | 2.71 | 2.73 | 2.80 | 0.80 | 2.80 | 2.83 | 2.85 | 1.12 | 3.12 | 3.09 | 2.73 | 1.09 | 3.08 | 3.02 | 2.73 | 1.35 | 0.256 | 0.061 | 0.353 | 0.581 | <0.001 | 0.315 | 0.514 | 0.926 |
| Protozoa (copy/mL) | 6.53 | 6.84 | 6.65 | 4.14 | 5.92 | 7.42 | 6.81 | 4.60 | 6.12 | 7.06 | 6.52 | 5.09 | 6.98 | 7.74 | 6.46 | 4.64 | 0.528 | 0.248 | 0.270 | 0.769 | <0.001 | 0.507 | 0.569 | 0.188 |
| Anaerobic fungi(copy/L) | 1.95 | 1.57 | 1.71 | -2.99 | 1.89 | 1.54 | 1.88 | -3.58 | 2.16 | 2.04 | 1.70 | -0.08 | 2.36 | 2.31 | 2.41 | -0.89 | 0.874 | 0.014 | 0.954 | 0.749 | <0.001 | 0.072 | 0.459 | 0.809 |
| **Bacterial diversity** |  |  |  |  |  |  |  |  |  |  |  |  |  |  |  |  |  |  |  |  |  |  |  |  |
| Richness | 405 | 410 | 389 | 497 | 352 | 326 | 337 | 463 | 492 | 485 | 473 | 560 | 429 | 402 | 425 | 505 | 24.46 | 0.017 | 0.045 | 0.906 | <0.001 | 0.524 | 0.394 | 0.950 |
| Shannon | 3.82 | 3.55 | 4.04 | 3.69 | 3.80 | 3.40 | 4.07 | 3.70 | 3.67 | 3.41 | 4.09 | 3.72 | 4.38 | 3.81 | 4.63 | 4.38 | 0.130 | 0.012 | 0.006 | 0.837 | <0.001 | 0.302 | 0.736 | 0.353 |
| Evenness | 0.64 | 0.63 | 0.62 | 0.71 | 0.61 | 0.59 | 0.59 | 0.62 | 0.65 | 0.66 | 0.66 | 0.73 | 0.61 | 0.62 | 0.62 | 0.70 | 0.021 | 0.018 | 0.004 | 0.760 | <0.001 | 0.197 | 0.712 | 0.311 |
| Simpson | 0.95 | 0.93 | 0.94 | 0.92 | 0.94 | 0.92 | 0.95 | 0.94 | 0.93 | 0.91 | 0.95 | 0.94 | 0.96 | 0.92 | 0.97 | 0.96 | 0.015 | 0.063 | 0.019 | 0.452 | 0.071 | 0.161 | 0.836 | 0.428 |
| Chao | 676 | 564 | 661 | 715 | 664 | 472 | 663 | 591 | 680 | 518 | 728 | 644 | 695 | 648 | 708 | 653 | 94.80 | 0.256 | 0.102 | 0.355 | 0.401 | 0.807 | 0.581 | 0.768 |
| Good's | 0.60 | 0.61 | 0.68 | 0.60 | 0.63 | 0.64 | 0.67 | 0.61 | 0.59 | 0.62 | 0.67 | 0.62 | 0.70 | 0.66 | 0.73 | 0.71 | 0.040 | 0.146 | 0.239 | 0.189 | 0.003 | 0.733 | 0.898 | 0.562 |

**Supplementary Table 5.** Abundance of the main bacteria at phyla. families and genera in the plant residue to describe the effect of the type of forage and vitamin E supplementation on the dynamics of plant colonization in the Rusitec system.

|  |  |  | **GRA-** | | | | **GRA+** | | | | **HAY-** | | | | **HAY+** | | | |  | **P-value** | | | | | | |
| --- | --- | --- | --- | --- | --- | --- | --- | --- | --- | --- | --- | --- | --- | --- | --- | --- | --- | --- | --- | --- | --- | --- | --- | --- | --- | --- |
| **Phylum** | **Family** | **Genus** | **2 h** | **4 h** | **8 h** | **24 h** | **2 h** | **4 h** | **8 h** | **24 h** | **2 h** | **4 h** | **8 h** | **24 h** | **2 h** | **4 h** | **8 h** | **24 h** | **SED^1^** | **F** | **V** | **T** | **FxV** | **FxT** | **VxT** | **FxVxT** |
| Actinobacteria |  |  | 2.07 | 1.97 | 1.54 | 2.02 | 1.99 | 1.73 | 1.26 | 1.79 | 2.76 | 2.58 | 2.31 | 2.28 | 2.67 | 2.50 | 2.11 | 1.95 | 0.170 | <0.001 | 0.04 | <0.001 | 0.838 | 0.01 | 0.635 | 0.86 |
|  | Coriobacteriaceae |  | 1.83 | 1.86 | 1.44 | 1.97 | 1.77 | 1.60 | 1.11 | 1.73 | 2.29 | 2.28 | 2.12 | 2.22 | 2.14 | 2.34 | 2.01 | 1.91 | 0.169 | <0.001 | 0.097 | <0.001 | 0.635 | 0.015 | 0.626 | 0.533 |
|  |  | Atopobium | 0.48 | 0.45 | 0.15 | 0.23 | 0.52 | 0.15 | 0.23 | 0.15 | 0.67 | 0.27 | 0.42 | 0.42 | 0.56 | 0.46 | 0.40 | 0.52 | 0.116 | 0.006 | 0.818 | 0.001 | 0.294 | 0.257 | 0.831 | 0.049 |
|  |  | Denitrobacterium | 0.19 | 0.15 | 0.00 | 0.15 | 0.08 | 0.08 | 0.00 | 0.15 | 0.35 | 0.37 | 0.23 | 0.35 | 0.33 | 0.30 | 0.27 | 0.19 | 0.149 | 0.008 | 0.42 | 0.408 | 1 | 0.751 | 0.812 | 0.765 |
|  |  | Olsenella | 1.77 | 1.84 | 1.41 | 1.95 | 1.73 | 1.57 | 1.06 | 1.68 | 2.24 | 2.26 | 2.08 | 2.17 | 2.10 | 2.31 | 1.98 | 1.80 | 0.178 | <0.001 | 0.097 | 0.002 | 0.677 | 0.015 | 0.541 | 0.491 |
|  |  | Slackia | 0.19 | 0.23 | 0.27 | 0.23 | 0.30 | 0.19 | 0.08 | 0.52 | 0.54 | 0.43 | 0.54 | 0.71 | 0.45 | 0.52 | 0.36 | 0.83 | 0.174 | 0.005 | 0.878 | 0.042 | 0.705 | 0.733 | 0.205 | 0.678 |
|  | Microbacteriaceae |  | 1.20 | 0.84 | 0.46 | 0.67 | 1.15 | 0.71 | 0.36 | 0.68 | 2.20 | 1.90 | 1.46 | 0.93 | 2.16 | 1.63 | 1.10 | 0.83 | 0.204 | <0.001 | 0.151 | <0.001 | 0.473 | 0.003 | 0.646 | 0.886 |
|  |  | Agreia | 0.85 | 0.39 | 0.15 | 0.35 | 0.77 | 0.38 | 0.15 | 0.42 | 1.61 | 1.29 | 0.97 | 0.50 | 1.62 | 1.04 | 0.60 | 0.60 | 0.148 | <0.001 | 0.492 | <0.001 | 0.524 | 0.001 | 0.278 | 0.35 |
|  |  | Clavibacter | 0.62 | 0.51 | 0.23 | 0.15 | 0.54 | 0.23 | 0.19 | 0.15 | 1.73 | 1.39 | 0.91 | 0.51 | 1.68 | 1.10 | 0.61 | 0.33 | 0.192 | <0.001 | 0.063 | <0.001 | 0.477 | 0.002 | 0.604 | 0.783 |
|  | Nocardiaceae |  | 0.99 | 0.71 | 0.33 | 0.50 | 0.94 | 0.60 | 0.35 | 0.19 | 1.92 | 1.72 | 1.22 | 0.38 | 2.00 | 1.38 | 0.90 | 0.39 | 0.135 | <0.001 | 0.191 | <0.001 | 0.876 | <0.001 | 0.334 | 0.094 |
|  |  | Rhodococcus | 0.97 | 0.69 | 0.33 | 0.50 | 0.91 | 0.58 | 0.35 | 0.19 | 1.88 | 1.67 | 1.18 | 0.36 | 1.96 | 1.35 | 0.87 | 0.38 | 0.127 | <0.001 | 0.209 | <0.001 | 0.909 | <0.001 | 0.348 | 0.081 |
| Bacteroidetes |  |  | 3.46 | 3.48 | 3.48 | 3.47 | 3.50 | 3.56 | 3.40 | 3.43 | 3.47 | 3.43 | 3.46 | 3.47 | 3.43 | 3.38 | 3.54 | 3.41 | 0.084 | 0.368 | 0.731 | 0.899 | 0.782 | 0.234 | 0.798 | 0.38 |
|  | Cytophagaceae |  | 2.66 | 2.06 | 1.65 | 1.08 | 2.58 | 2.03 | 1.52 | 1.03 | 2.62 | 2.12 | 1.81 | 1.05 | 2.47 | 1.88 | 1.54 | 0.81 | 0.190 | 0.684 | 0.145 | <0.001 | 0.423 | 0.566 | 0.877 | 0.919 |
|  |  | Dyadobacter | 2.04 | 1.39 | 0.83 | 0.81 | 1.91 | 1.39 | 0.80 | 0.70 | 2.30 | 1.81 | 1.58 | 0.78 | 2.15 | 1.61 | 1.32 | 0.67 | 0.191 | 0.006 | 0.162 | <0.001 | 0.49 | 0.037 | 0.959 | 0.816 |
|  |  | Hymenobacter | 1.68 | 1.19 | 0.58 | 0.23 | 1.77 | 0.98 | 0.50 | 0.29 | 1.88 | 1.49 | 0.98 | 0.30 | 1.82 | 1.06 | 0.78 | 0.19 | 0.127 | 0.042 | 0.127 | <0.001 | 0.258 | 0.085 | 0.07 | 0.945 |
|  |  | Leadbetterella | 2.18 | 1.77 | 1.44 | 0.54 | 2.24 | 1.74 | 1.32 | 0.75 | 1.37 | 0.85 | 0.82 | 0.47 | 0.83 | 0.61 | 0.67 | 0.08 | 0.247 | <0.001 | 0.27 | <0.001 | 0.201 | 0.024 | 0.927 | 0.554 |
|  |  | Spirosoma | 1.21 | 0.54 | 0.15 | 0.12 | 1.09 | 0.35 | 0.08 | 0.08 | 1.82 | 1.37 | 0.97 | 0.19 | 1.77 | 1.20 | 0.65 | 0.31 | 0.149 | <0.001 | 0.299 | <0.001 | 0.986 | <0.001 | 0.364 | 0.532 |
|  | Flavobacteriaceae |  | 2.60 | 2.21 | 1.74 | 1.85 | 2.53 | 2.13 | 1.60 | 2.08 | 2.72 | 2.47 | 2.07 | 1.70 | 2.66 | 2.34 | 2.04 | 2.07 | 0.130 | 0.038 | 0.858 | <0.001 | 0.716 | 0.013 | 0.015 | 0.83 |
|  |  | Chryseobacterium | 1.39 | 1.14 | 0.54 | 0.51 | 1.44 | 0.94 | 0.69 | 0.49 | 1.88 | 1.63 | 1.33 | 0.38 | 1.79 | 1.35 | 1.07 | 0.52 | 0.161 | <0.001 | 0.375 | <0.001 | 0.42 | 0.005 | 0.31 | 0.361 |
|  |  | Costertonia | 1.08 | 1.35 | 1.31 | 1.61 | 1.11 | 1.52 | 1.16 | 1.97 | 1.18 | 1.73 | 1.56 | 1.45 | 1.30 | 1.88 | 1.78 | 2.00 | 0.260 | 0.237 | 0.327 | 0.013 | 0.66 | 0.242 | 0.329 | 0.693 |
|  |  | Flavobacterium | 2.44 | 1.99 | 1.37 | 1.16 | 2.40 | 1.83 | 1.16 | 0.81 | 2.53 | 2.18 | 1.59 | 0.71 | 2.45 | 1.93 | 1.28 | 0.61 | 0.233 | 0.882 | 0.091 | <0.001 | 0.985 | 0.174 | 0.703 | 0.724 |
|  | Porphyromonadaceae |  | 1.26 | 1.40 | 1.24 | 1.25 | 1.32 | 1.42 | 1.08 | 1.51 | 1.58 | 1.67 | 1.63 | 1.08 | 1.60 | 1.79 | 1.67 | 1.36 | 0.149 | 0.011 | 0.305 | 0.015 | 0.65 | 0.002 | 0.184 | 0.836 |
|  | Prevotellaceae |  | 3.23 | 3.40 | 3.43 | 3.40 | 3.32 | 3.50 | 3.36 | 3.29 | 3.07 | 3.24 | 3.35 | 3.39 | 3.10 | 3.21 | 3.44 | 3.27 | 0.112 | 0.008 | 0.903 | 0.012 | 0.901 | 0.147 | 0.391 | 0.562 |
|  |  | Hallella | 1.18 | 1.21 | 1.03 | 1.38 | 1.27 | 1.25 | 1.15 | 1.40 | 1.88 | 1.86 | 1.77 | 1.32 | 1.83 | 1.85 | 1.73 | 1.43 | 0.178 | <0.001 | 0.707 | 0.193 | 0.702 | 0.003 | 0.975 | 0.826 |
|  |  | Paraprevotella | 0.44 | 0.72 | 0.73 | 0.84 | 0.51 | 0.86 | 0.89 | 0.71 | 1.11 | 1.30 | 1.24 | 1.12 | 1.34 | 1.48 | 1.77 | 1.35 | 0.153 | <0.001 | 0.161 | 0.004 | 0.333 | 0.275 | 0.293 | 0.64 |
|  |  | Prevotella | 3.20 | 3.35 | 3.35 | 3.33 | 3.28 | 3.46 | 3.29 | 3.22 | 2.88 | 3.02 | 3.11 | 3.29 | 2.93 | 2.97 | 3.13 | 3.10 | 0.121 | <0.001 | 0.541 | 0.064 | 0.442 | 0.07 | 0.294 | 0.705 |
|  | Rikenellaceae |  | 1.54 | 1.45 | 1.28 | 1.66 | 1.58 | 1.58 | 1.40 | 1.84 | 1.78 | 1.58 | 1.55 | 1.70 | 1.70 | 1.56 | 1.50 | 1.93 | 0.113 | 0.053 | 0.24 | <0.001 | 0.393 | 0.449 | 0.238 | 0.645 |
|  |  | Rikenella | 1.52 | 1.40 | 1.24 | 1.59 | 1.53 | 1.51 | 1.36 | 1.75 | 1.70 | 1.46 | 1.47 | 1.63 | 1.61 | 1.47 | 1.44 | 1.83 | 0.118 | 0.124 | 0.264 | 0.001 | 0.455 | 0.498 | 0.319 | 0.715 |
|  | Sphingobacteriaceae |  | 1.90 | 1.63 | 1.25 | 1.00 | 1.92 | 1.49 | 1.14 | 0.89 | 2.47 | 2.20 | 1.92 | 0.82 | 2.29 | 1.98 | 1.56 | 0.77 | 0.205 | 0.001 | 0.1 | <0.001 | 0.484 | 0.02 | 0.702 | 0.769 |
|  |  | Pedobacter | 1.79 | 1.45 | 1.08 | 0.87 | 1.77 | 1.35 | 1.02 | 0.67 | 2.40 | 2.11 | 1.79 | 0.74 | 2.22 | 1.87 | 1.47 | 0.70 | 0.191 | <0.001 | 0.072 | <0.001 | 0.517 | 0.019 | 0.855 | 0.602 |
|  |  | Sphingobacterium | 1.03 | 1.09 | 0.74 | 0.44 | 1.22 | 0.83 | 0.50 | 0.53 | 1.39 | 1.22 | 1.11 | 0.35 | 1.22 | 1.13 | 0.69 | 0.19 | 0.231 | 0.185 | 0.135 | <0.001 | 0.392 | 0.178 | 0.416 | 0.587 |
| Elusimicrobia | Candidatus Endomicrobium | Candidatus Endomicrobium | 0.69 | 0.80 | 0.73 | 0.95 | 0.64 | 0.75 | 0.40 | 0.97 | 1.52 | 1.33 | 1.27 | 0.97 | 1.63 | 1.48 | 1.32 | 1.27 | 0.204 | <0.001 | 0.826 | 0.282 | 0.279 | 0.011 | 0.514 | 0.914 |
| Firmicutes |  |  | 3.89 | 3.90 | 3.90 | 3.89 | 3.90 | 3.86 | 3.93 | 3.92 | 3.84 | 3.89 | 3.89 | 3.88 | 3.86 | 3.91 | 3.86 | 3.90 | 0.038 | 0.187 | 0.546 | 0.531 | 0.984 | 0.377 | 0.813 | 0.458 |
|  | Clostridiaceae 1 |  | 0.96 | 1.06 | 0.92 | 0.84 | 0.81 | 1.12 | 0.64 | 0.78 | 1.44 | 1.54 | 1.43 | 0.97 | 1.88 | 2.32 | 1.92 | 1.03 | 0.335 | 0.016 | 0.483 | 0.039 | 0.263 | 0.183 | 0.52 | 0.608 |
|  |  | Clostridium sensu stricto | 0.80 | 0.98 | 0.87 | 0.54 | 0.69 | 0.98 | 0.49 | 0.71 | 1.39 | 1.22 | 1.22 | 0.51 | 1.86 | 2.31 | 1.91 | 0.91 | 0.361 | 0.047 | 0.338 | 0.024 | 0.228 | 0.144 | 0.552 | 0.477 |
|  | Clostridiales_Incertae Sedis XIII |  | 1.62 | 1.57 | 1.42 | 1.71 | 1.70 | 1.59 | 1.34 | 1.74 | 2.01 | 1.84 | 1.78 | 1.74 | 2.10 | 1.98 | 1.85 | 1.89 | 0.106 | <0.001 | 0.147 | 0.002 | 0.22 | 0.025 | 0.722 | 0.85 |
|  |  | Anaerovorax | 1.13 | 1.12 | 1.00 | 1.35 | 1.22 | 1.20 | 0.91 | 1.37 | 1.64 | 1.43 | 1.36 | 1.37 | 1.67 | 1.56 | 1.46 | 1.54 | 0.116 | <0.001 | 0.24 | 0.004 | 0.425 | 0.018 | 0.766 | 0.634 |
|  |  | Mogibacterium | 1.28 | 1.19 | 1.07 | 1.30 | 1.37 | 1.16 | 0.95 | 1.28 | 1.60 | 1.45 | 1.44 | 1.32 | 1.70 | 1.66 | 1.47 | 1.44 | 0.119 | <0.001 | 0.342 | 0.008 | 0.191 | 0.057 | 0.574 | 0.724 |
|  | Eubacteriaceae |  | 2.58 | 2.61 | 2.48 | 3.05 | 2.72 | 2.60 | 2.50 | 2.93 | 2.19 | 2.19 | 2.16 | 2.74 | 2.44 | 2.42 | 2.26 | 2.43 | 0.114 | 0.016 | 0.735 | <0.001 | 0.782 | 0.573 | 0.031 | 0.295 |
|  |  | Eubacterium | 2.10 | 2.19 | 2.03 | 2.81 | 2.24 | 2.10 | 2.01 | 2.45 | 2.13 | 2.15 | 2.11 | 2.53 | 2.35 | 2.36 | 2.11 | 2.35 | 0.142 | 0.839 | 0.894 | <0.001 | 0.431 | 0.16 | 0.036 | 0.717 |
|  |  | Pseudoramibacter | 2.33 | 2.35 | 2.21 | 2.18 | 2.52 | 2.36 | 2.31 | 2.60 | 1.11 | 0.98 | 0.97 | 2.11 | 1.04 | 1.03 | 0.97 | 1.10 | 0.312 | 0.002 | 0.897 | 0.153 | 0.44 | 0.239 | 0.468 | 0.149 |
|  | Lachnospiraceae |  | 3.31 | 3.57 | 3.55 | 3.45 | 3.54 | 3.58 | 3.54 | 3.46 | 3.20 | 3.34 | 3.38 | 3.42 | 3.27 | 3.49 | 3.56 | 3.46 | 0.107 | 0.077 | 0.159 | 0.024 | 0.687 | 0.34 | 0.606 | 0.385 |
|  |  | Blautia | 0.66 | 0.69 | 0.66 | 1.19 | 0.61 | 0.88 | 0.86 | 1.39 | 1.01 | 1.12 | 1.06 | 1.24 | 0.87 | 1.11 | 1.21 | 1.44 | 0.171 | 0.005 | 0.23 | <0.001 | 0.58 | 0.26 | 0.32 | 0.915 |
|  |  | Butyrivibrio | 2.73 | 3.05 | 2.98 | 2.71 | 2.94 | 3.03 | 2.91 | 2.71 | 2.44 | 2.57 | 2.66 | 2.74 | 2.52 | 2.81 | 2.96 | 2.88 | 0.130 | 0.048 | 0.206 | 0.016 | 0.343 | 0.011 | 0.853 | 0.284 |
|  |  | Clostridium XlVa | 1.42 | 1.47 | 1.21 | 1.38 | 1.85 | 1.50 | 1.22 | 1.28 | 1.48 | 1.77 | 1.76 | 1.61 | 1.82 | 1.93 | 1.78 | 1.70 | 0.133 | <0.001 | 0.015 | 0.042 | 0.456 | 0.016 | 0.051 | 0.578 |
|  |  | Coprococcus | 1.25 | 1.46 | 1.61 | 1.29 | 1.37 | 1.47 | 1.56 | 1.49 | 1.16 | 1.12 | 1.34 | 1.40 | 1.15 | 1.35 | 1.60 | 1.40 | 0.137 | 0.287 | 0.41 | 0.011 | 0.829 | 0.339 | 0.879 | 0.209 |
|  |  | Lachnobacterium | 1.48 | 1.64 | 1.47 | 1.24 | 1.66 | 1.69 | 1.49 | 1.31 | 1.34 | 1.49 | 1.42 | 1.28 | 1.49 | 1.55 | 1.53 | 1.24 | 0.123 | 0.275 | 0.307 | 0.001 | 0.938 | 0.438 | 0.568 | 0.794 |
|  |  | Lachnospiracea_incertae_sedis | 2.08 | 2.67 | 2.92 | 2.35 | 2.53 | 2.75 | 3.11 | 2.37 | 1.84 | 1.97 | 2.08 | 2.33 | 1.76 | 2.07 | 2.33 | 2.26 | 0.142 | <0.001 | 0.261 | <0.001 | 0.496 | 0.001 | 0.349 | 0.192 |
|  |  | Oribacterium | 1.02 | 0.93 | 0.66 | 0.79 | 1.07 | 1.02 | 0.72 | 0.67 | 1.19 | 1.55 | 1.53 | 0.98 | 1.35 | 1.59 | 1.36 | 0.99 | 0.182 | <0.001 | 0.857 | 0.005 | 0.913 | 0.038 | 0.637 | 0.625 |
|  |  | Pseudobutyrivibrio | 2.33 | 2.78 | 2.61 | 2.01 | 2.63 | 2.74 | 2.48 | 1.95 | 2.20 | 2.52 | 2.59 | 2.20 | 2.24 | 2.72 | 2.82 | 2.26 | 0.169 | 0.987 | 0.39 | <0.001 | 0.532 | 0.047 | 0.653 | 0.305 |
|  |  | Roseburia | 1.53 | 2.01 | 2.03 | 2.15 | 1.75 | 1.85 | 1.88 | 2.14 | 1.76 | 2.01 | 2.17 | 2.07 | 1.65 | 1.98 | 2.21 | 2.24 | 0.151 | 0.169 | 0.963 | <0.001 | 0.7 | 0.471 | 0.567 | 0.274 |
|  | Lactobacillaceae | Lactobacillus | 3.06 | 2.90 | 2.51 | 2.73 | 2.94 | 2.53 | 2.31 | 2.80 | 2.67 | 2.82 | 3.05 | 2.48 | 2.71 | 2.55 | 2.66 | 2.39 | 0.176 | 0.667 | 0.229 | 0.062 | 0.945 | 0.002 | 0.2 | 0.598 |
|  | Peptococcaceae 1 |  | 1.76 | 1.63 | 1.43 | 1.76 | 1.64 | 1.65 | 1.25 | 1.86 | 2.29 | 2.00 | 1.84 | 1.80 | 2.26 | 2.03 | 1.84 | 1.89 | 0.131 | <0.001 | 0.878 | <0.001 | 0.63 | 0.005 | 0.41 | 0.802 |
|  |  | Dehalobacter | 1.59 | 1.46 | 1.28 | 1.63 | 1.45 | 1.48 | 1.11 | 1.72 | 2.13 | 1.86 | 1.68 | 1.63 | 2.10 | 1.88 | 1.67 | 1.72 | 0.142 | <0.001 | 0.802 | 0.001 | 0.587 | 0.007 | 0.481 | 0.833 |
|  | Ruminococcaceae |  | 3.00 | 3.17 | 3.43 | 3.13 | 3.12 | 3.19 | 3.61 | 3.25 | 2.72 | 2.55 | 2.57 | 3.28 | 2.73 | 2.57 | 3.01 | 3.43 | 0.172 | <0.001 | 0.092 | 0.001 | 0.777 | <0.001 | 0.358 | 0.63 |
|  |  | Acetanaerobacterium | 1.27 | 1.15 | 0.89 | 0.31 | 1.43 | 1.18 | 0.87 | 0.42 | 1.26 | 1.00 | 0.93 | 0.57 | 1.11 | 0.91 | 0.77 | 0.56 | 0.191 | 0.663 | 0.901 | <0.001 | 0.48 | 0.168 | 0.857 | 0.926 |
|  |  | Acetivibrio | 0.95 | 0.94 | 0.52 | 0.87 | 0.97 | 0.86 | 0.75 | 1.03 | 0.99 | 0.55 | 0.70 | 0.70 | 0.94 | 0.54 | 0.44 | 0.89 | 0.180 | 0.111 | 0.773 | 0.005 | 0.478 | 0.238 | 0.546 | 0.391 |
|  |  | Clostridium IV | 1.71 | 1.82 | 1.75 | 1.82 | 1.77 | 1.75 | 1.64 | 2.05 | 1.88 | 1.59 | 1.55 | 1.77 | 1.66 | 1.63 | 1.65 | 1.96 | 0.167 | 0.326 | 0.723 | 0.046 | 0.953 | 0.623 | 0.332 | 0.451 |
|  |  | Ethanoligenens | 0.41 | 0.30 | 0.00 | 0.00 | 0.61 | 0.35 | 0.00 | 0.35 | 0.58 | 0.15 | 0.15 | 0.15 | 0.61 | 0.23 | 0.15 | 0.15 | 0.128 | 0.724 | 0.168 | <0.001 | 0.32 | 0.172 | 0.509 | 0.405 |
|  |  | Flavonifractor | 1.29 | 1.17 | 0.93 | 0.74 | 1.33 | 1.23 | 1.08 | 0.96 | 0.88 | 0.54 | 0.45 | 0.72 | 0.58 | 0.30 | 0.60 | 0.62 | 0.190 | 0.001 | 0.994 | 0.049 | 0.278 | 0.044 | 0.418 | 0.704 |
|  |  | Oscillibacter | 1.45 | 1.38 | 1.31 | 0.76 | 1.45 | 1.17 | 1.24 | 0.85 | 1.12 | 0.96 | 1.05 | 0.82 | 1.33 | 1.05 | 1.07 | 0.91 | 0.236 | 0.012 | 0.624 | 0.013 | 0.185 | 0.426 | 0.723 | 0.802 |
|  |  | Ruminococcus | 2.78 | 3.07 | 3.40 | 3.04 | 2.99 | 3.11 | 3.59 | 3.14 | 2.12 | 2.22 | 2.33 | 3.22 | 2.23 | 2.22 | 2.90 | 3.36 | 0.198 | <0.001 | 0.083 | <0.001 | 0.726 | <0.001 | 0.328 | 0.562 |
|  | Streptococcaceae | Streptococcus | 2.41 | 2.47 | 2.07 | 2.13 | 2.56 | 2.44 | 1.84 | 2.01 | 3.25 | 3.52 | 3.39 | 2.09 | 3.33 | 3.37 | 3.01 | 2.23 | 0.251 | <0.001 | 0.316 | <0.001 | 0.893 | 0.001 | 0.371 | 0.791 |
|  | Veillonellaceae |  | 2.24 | 2.20 | 1.85 | 1.57 | 2.62 | 2.26 | 1.80 | 1.68 | 2.43 | 2.46 | 2.23 | 1.88 | 2.48 | 2.37 | 2.28 | 1.91 | 0.138 | 0.003 | 0.261 | <0.001 | 0.324 | 0.062 | 0.329 | 0.438 |
|  |  | Anaerovibrio | 1.18 | 1.32 | 1.06 | 1.02 | 1.41 | 1.44 | 0.93 | 1.14 | 1.24 | 1.30 | 1.28 | 1.02 | 1.39 | 1.36 | 1.37 | 1.23 | 0.137 | 0.099 | 0.045 | 0.013 | 0.657 | 0.084 | 0.375 | 0.573 |
|  |  | Selenomonas | 2.08 | 2.03 | 1.68 | 1.23 | 2.53 | 2.11 | 1.68 | 1.35 | 2.28 | 2.31 | 2.06 | 1.67 | 2.25 | 2.22 | 2.15 | 1.66 | 0.169 | 0.006 | 0.296 | <0.001 | 0.237 | 0.055 | 0.593 | 0.391 |
| Proteobacteria |  |  | 1.98 | 1.49 | 1.08 | 1.19 | 1.91 | 1.40 | 0.97 | 1.22 | 2.56 | 2.18 | 1.87 | 1.03 | 2.44 | 1.92 | 1.60 | 1.21 | 0.201 | <0.001 | 0.256 | <0.001 | 0.721 | 0.009 | 0.395 | 0.733 |
|  | Comamonadaceae |  | 1.37 | 0.87 | 0.39 | 0.46 | 1.34 | 0.69 | 0.38 | 0.60 | 1.74 | 1.36 | 1.06 | 0.42 | 1.64 | 1.07 | 0.63 | 0.41 | 0.165 | 0.002 | 0.117 | <0.001 | 0.197 | 0.009 | 0.245 | 0.66 |
|  |  | Variovorax | 1.23 | 0.72 | 0.35 | 0.43 | 1.17 | 0.54 | 0.38 | 0.47 | 1.56 | 1.22 | 0.96 | 0.40 | 1.51 | 0.95 | 0.56 | 0.37 | 0.179 | 0.002 | 0.113 | <0.001 | 0.309 | 0.044 | 0.514 | 0.558 |
|  | Oxalobacteraceae |  | 1.04 | 0.60 | 0.23 | 0.00 | 0.92 | 0.44 | 0.12 | 0.40 | 1.77 | 1.42 | 1.09 | 0.23 | 1.73 | 1.16 | 0.54 | 0.27 | 0.163 | <0.001 | 0.234 | <0.001 | 0.208 | <0.001 | 0.024 | 0.366 |
|  | Pseudomonadaceae |  | 1.26 | 0.80 | 0.52 | 0.29 | 1.17 | 0.73 | 0.37 | 0.23 | 1.69 | 1.10 | 0.66 | 0.15 | 1.42 | 0.90 | 0.61 | 0.08 | 0.194 | 0.085 | 0.166 | <0.001 | 0.751 | 0.101 | 0.928 | 0.873 |
|  | Sutterellaceae | Sutterella | 0.19 | 0.23 | 0.15 | 0.00 | 0.15 | 0.30 | 0.00 | 0.00 | 0.40 | 0.50 | 0.46 | 0.08 | 0.61 | 0.59 | 0.53 | 0.00 | 0.128 | 0.003 | 0.752 | <0.001 | 0.463 | 0.057 | 0.524 | 0.477 |
| Spirochaetes | Spirochaetaceae |  | 0.88 | 0.96 | 1.17 | 1.71 | 0.81 | 1.13 | 0.69 | 1.56 | 1.89 | 1.87 | 1.88 | 2.10 | 1.99 | 2.01 | 2.04 | 2.27 | 0.285 | <0.001 | 0.971 | 0.014 | 0.203 | 0.264 | 0.655 | 0.605 |
|  |  | Treponema | 0.84 | 0.95 | 1.15 | 1.69 | 0.74 | 1.09 | 0.69 | 1.53 | 1.76 | 1.79 | 1.85 | 2.06 | 1.90 | 1.94 | 2.00 | 2.23 | 0.275 | <0.001 | 0.959 | 0.007 | 0.19 | 0.266 | 0.66 | 0.649 |
| Tenericutes | Acholeplasmataceae |  | 0.99 | 1.26 | 1.40 | 1.68 | 0.97 | 1.41 | 1.07 | 1.48 | 1.56 | 1.63 | 1.92 | 1.60 | 1.64 | 1.73 | 1.93 | 1.88 | 0.292 | <0.001 | 0.924 | 0.125 | 0.201 | 0.266 | 0.685 | 0.693 |
|  |  | Acholeplasma | 0.64 | 0.48 | 1.00 | 1.51 | 0.55 | 0.95 | 0.65 | 1.25 | 0.89 | 1.01 | 1.36 | 1.42 | 0.97 | 0.96 | 1.36 | 1.71 | 0.307 | 0.011 | 0.928 | 0.003 | 0.504 | 0.618 | 0.58 | 0.33 |
|  |  | Anaeroplasma | 0.77 | 1.16 | 1.19 | 1.18 | 0.86 | 1.17 | 0.88 | 1.15 | 1.42 | 1.50 | 1.74 | 1.13 | 1.51 | 1.65 | 1.79 | 1.38 | 0.269 | <0.001 | 0.69 | 0.195 | 0.299 | 0.125 | 0.702 | 0.821 |
| Verrucomicrobia | Subdivision5_genera_incertae_sedis |  | 0.63 | 0.69 | 0.53 | 0.91 | 0.83 | 0.53 | 0.27 | 1.02 | 1.37 | 1.04 | 0.97 | 1.04 | 1.23 | 1.07 | 0.77 | 1.29 | 0.190 | 0.004 | 0.838 | <0.001 | 0.951 | 0.262 | 0.209 | 0.473 |
| Unclassified |  |  | 0.54 | 0.51 | 0.27 | 0.68 | 0.51 | 0.52 | 0.19 | 0.80 | 1.24 | 1.02 | 0.83 | 0.81 | 1.17 | 1.10 | 0.85 | 0.95 | 0.174 | <0.001 | 0.72 | 0.006 | 0.771 | 0.026 | 0.685 | 0.969 |

^1^Standard error of the difference for the interaction between the type of forage (F). vitamin E supplementation at 50 IU/d (V) and incubation time (T) (n=4). The total number of reads per sample was log-transformed and minor genera (<0.01%) were discarded.

**Supplementary Table 6.** Abundance of the main bacteria at phyla. families and genera in liquid phase to describe the effect of the type of forage and vitamin E supplementation on the dynamics of nutrient utilization in the Rusitec system.

|  |  |  | **GRA-** | | | | **GRA+** | | | | **HAY-** | | | | **HAY+** | | | |  | **P-value** | | | | | | |
| --- | --- | --- | --- | --- | --- | --- | --- | --- | --- | --- | --- | --- | --- | --- | --- | --- | --- | --- | --- | --- | --- | --- | --- | --- | --- | --- |
| **Phylum** | **Family** | **Genus** | **2 h** | **4 h** | **8 h** | **24 h** | **2 h** | **4 h** | **8 h** | **24 h** | **2 h** | **4 h** | **8 h** | **24 h** | **2 h** | **4 h** | **8 h** | **24 h** | **SED^1^** | **F** | **V** | **T** | **FxV** | **FxT** | **VxT** | **FxVxT** |
| Actinobacteria |  |  | 1.88 | 1.60 | 1.23 | 1.85 | 1.71 | 1.41 | 0.97 | 1.73 | 1.98 | 1.87 | 1.71 | 2.08 | 1.93 | 1.81 | 1.68 | 2.22 | 0.206 | 0.001 | 0.267 | <0.001 | 0.277 | 0.239 | 0.788 | 0.93 |
|  | Coriobacteriaceae |  | 1.87 | 1.54 | 1.23 | 1.84 | 1.70 | 1.40 | 0.96 | 1.63 | 1.98 | 1.86 | 1.71 | 2.08 | 1.92 | 1.80 | 1.68 | 2.22 | 0.205 | <0.001 | 0.228 | <0.001 | 0.233 | 0.231 | 0.885 | 0.815 |
|  |  | Atopobium | 0.85 | 0.66 | 0.70 | 1.15 | 0.60 | 0.70 | 0.44 | 0.93 | 0.93 | 1.02 | 1.03 | 1.13 | 0.87 | 0.91 | 1.01 | 1.29 | 0.118 | 0.014 | 0.336 | <0.001 | 0.359 | 0.085 | 0.537 | 0.168 |
|  |  | Denitrobacterium | 0.82 | 0.50 | 0.35 | 0.70 | 0.90 | 0.51 | 0.29 | 0.65 | 1.08 | 0.66 | 0.52 | 0.63 | 0.71 | 0.57 | 0.48 | 0.89 | 0.365 | 0.485 | 0.82 | 0.135 | 0.854 | 0.856 | 0.746 | 0.592 |
|  |  | Olsenella | 1.59 | 1.40 | 0.83 | 1.27 | 1.29 | 1.16 | 0.61 | 1.09 | 1.56 | 1.63 | 1.43 | 1.73 | 1.58 | 1.64 | 1.43 | 1.94 | 0.172 | 0.003 | 0.463 | <0.001 | 0.251 | 0.018 | 0.717 | 0.906 |
|  |  | Slackia | 0.69 | 0.43 | 0.45 | 1.40 | 0.44 | 0.57 | 0.44 | 1.22 | 0.60 | 1.01 | 0.89 | 1.58 | 0.71 | 0.84 | 0.82 | 1.58 | 0.127 | 0.013 | 0.583 | <0.001 | 0.823 | 0.062 | 0.898 | 0.082 |
|  | Microbacteriaceae |  | 0.08 | 0.34 | 0.00 | 0.19 | 0.08 | 0.00 | 0.00 | 0.15 | 0.08 | 0.00 | 0.00 | 0.21 | 0.08 | 0.08 | 0.00 | 0.00 | 0.158 | 0.425 | 0.304 | 0.338 | 0.615 | 0.697 | 0.615 | 0.306 |
|  | Nocardiaceae |  | 0.00 | 0.27 | 0.00 | 0.00 | 0.08 | 0.00 | 0.00 | 0.00 | 0.00 | 0.08 | 0.08 | 0.08 | 0.08 | 0.00 | 0.00 | 0.00 | 0.112 | 0.901 | 0.342 | 0.472 | 0.901 | 0.472 | 0.223 | 0.472 |
| Bacteroidetes |  |  | 3.75 | 3.66 | 3.73 | 3.54 | 3.73 | 3.73 | 3.79 | 3.58 | 3.64 | 3.72 | 3.73 | 3.62 | 3.68 | 3.75 | 3.74 | 3.56 | 0.061 | 0.756 | 0.446 | <0.001 | 0.542 | 0.202 | 0.664 | 0.561 |
|  | Cytophagaceae |  | 0.15 | 0.55 | 0.15 | 0.15 | 0.00 | 0.00 | 0.00 | 0.00 | 0.08 | 0.00 | 0.23 | 0.00 | 0.08 | 0.12 | 0.00 | 0.00 | 0.209 | 0.223 | 0.019 | 0.521 | 0.046 | 0.508 | 0.693 | 0.32 |
|  | Flavobacteriaceae |  | 1.75 | 1.71 | 1.67 | 2.09 | 1.38 | 1.26 | 1.46 | 1.88 | 1.81 | 1.81 | 1.84 | 2.17 | 1.86 | 1.84 | 2.04 | 2.18 | 0.138 | 0.008 | 0.205 | <0.001 | 0.056 | 0.532 | 0.479 | 0.769 |
|  |  | Chryseobacterium | 0.08 | 0.30 | 0.08 | 0.00 | 0.00 | 0.08 | 0.08 | 0.00 | 0.00 | 0.00 | 0.12 | 0.25 | 0.00 | 0.08 | 0.08 | 0.00 | 0.157 | 0.857 | 0.271 | 0.648 | 0.857 | 0.396 | 0.875 | 0.396 |
|  |  | Costertonia | 1.54 | 1.43 | 1.48 | 1.63 | 1.24 | 0.97 | 1.24 | 1.54 | 1.67 | 1.62 | 1.64 | 1.93 | 1.83 | 1.78 | 1.99 | 2.02 | 0.152 | 0.001 | 0.677 | 0.005 | 0.037 | 0.724 | 0.52 | 0.432 |
|  |  | Flavobacterium | 0.42 | 0.71 | 0.12 | 0.08 | 0.39 | 0.37 | 0.19 | 0.00 | 0.15 | 0.08 | 0.30 | 0.00 | 0.08 | 0.19 | 0.12 | 0.00 | 0.202 | 0.058 | 0.436 | 0.059 | 0.716 | 0.144 | 0.906 | 0.333 |
|  | Porphyromonadaceae |  | 1.17 | 1.30 | 1.30 | 1.56 | 1.05 | 1.17 | 1.23 | 1.53 | 1.58 | 1.81 | 1.83 | 1.79 | 1.51 | 1.59 | 1.78 | 1.82 | 0.110 | <0.001 | 0.361 | <0.001 | 0.929 | 0.112 | 0.469 | 0.847 |
|  | Prevotellaceae |  | 3.71 | 3.62 | 3.70 | 3.36 | 3.71 | 3.71 | 3.77 | 3.48 | 3.57 | 3.66 | 3.68 | 3.46 | 3.63 | 3.72 | 3.70 | 3.41 | 0.072 | 0.276 | 0.111 | <0.001 | 0.383 | 0.237 | 0.78 | 0.422 |
|  |  | Hallella | 1.31 | 1.30 | 1.29 | 1.90 | 1.12 | 1.19 | 1.22 | 1.77 | 2.01 | 2.00 | 2.12 | 2.32 | 1.59 | 1.72 | 1.83 | 2.13 | 0.139 | 0.004 | 0.201 | <0.001 | 0.591 | 0.154 | 0.68 | 0.884 |
|  |  | Paraprevotella | 0.30 | 0.24 | 0.39 | 0.52 | 0.08 | 0.12 | 0.19 | 0.42 | 0.95 | 1.00 | 0.90 | 1.01 | 1.07 | 0.94 | 0.95 | 0.95 | 0.129 | <0.001 | 0.281 | 0.111 | 0.197 | 0.082 | 0.992 | 0.574 |
|  |  | Prevotella | 3.70 | 3.60 | 3.68 | 3.26 | 3.70 | 3.70 | 3.75 | 3.43 | 3.52 | 3.62 | 3.63 | 3.34 | 3.60 | 3.69 | 3.66 | 3.31 | 0.084 | 0.077 | 0.072 | <0.001 | 0.45 | 0.332 | 0.805 | 0.402 |
|  | Rikenellaceae |  | 1.98 | 1.95 | 1.90 | 2.43 | 1.74 | 1.70 | 1.52 | 2.12 | 2.18 | 2.14 | 2.07 | 2.26 | 1.94 | 1.79 | 1.74 | 2.22 | 0.108 | 0.029 | <0.001 | <0.001 | 0.574 | 0.149 | 0.357 | 0.373 |
|  |  | Rikenella | 1.96 | 1.93 | 1.86 | 2.41 | 1.71 | 1.67 | 1.47 | 2.10 | 2.14 | 2.08 | 2.04 | 2.23 | 1.89 | 1.75 | 1.68 | 2.20 | 0.111 | 0.053 | <0.001 | <0.001 | 0.563 | 0.181 | 0.322 | 0.44 |
|  | Sphingobacteriaceae |  | 0.39 | 0.75 | 0.27 | 0.23 | 0.50 | 0.39 | 0.19 | 0.12 | 0.00 | 0.08 | 0.37 | 0.08 | 0.08 | 0.08 | 0.08 | 0.00 | 0.175 | 0.016 | 0.328 | 0.157 | 0.842 | 0.067 | 0.341 | 0.379 |
|  |  | Pedobacter | 0.27 | 0.55 | 0.15 | 0.08 | 0.21 | 0.31 | 0.08 | 0.00 | 0.00 | 0.08 | 0.30 | 0.00 | 0.08 | 0.08 | 0.08 | 0.00 | 0.182 | 0.09 | 0.307 | 0.136 | 0.608 | 0.144 | 0.721 | 0.667 |
|  |  | Sphingobacterium | 0.15 | 0.51 | 0.15 | 0.19 | 0.35 | 0.12 | 0.15 | 0.12 | 0.00 | 0.00 | 0.08 | 0.08 | 0.00 | 0.00 | 0.00 | 0.00 | 0.125 | 0.002 | 0.291 | 0.563 | 0.752 | 0.291 | 0.193 | 0.17 |
| Elusimicrobia | Candidatus Endomicrobium | Candidatus Endomicrobium | 0.19 | 0.39 | 0.33 | 0.61 | 0.12 | 0.00 | 0.23 | 0.43 | 0.56 | 0.60 | 0.58 | 1.25 | 0.37 | 0.31 | 0.63 | 0.85 | 0.138 | <0.001 | 0.027 | <0.001 | 0.879 | 0.251 | 0.157 | 0.409 |
| Firmicutes |  |  | 3.74 | 3.78 | 3.75 | 3.87 | 3.75 | 3.72 | 3.69 | 3.86 | 3.80 | 3.75 | 3.75 | 3.80 | 3.78 | 3.72 | 3.73 | 3.85 | 0.044 | 0.9 | 0.365 | <0.001 | 0.53 | 0.244 | 0.383 | 0.638 |
|  | Clostridiaceae 1 |  | 0.79 | 0.96 | 0.88 | 1.28 | 0.48 | 0.74 | 0.65 | 1.20 | 0.95 | 0.80 | 0.85 | 1.62 | 0.93 | 1.08 | 1.45 | 2.42 | 0.193 | 0.059 | 0.589 | <0.001 | 0.117 | 0.025 | 0.09 | 0.366 |
|  |  | Clostridium sensu stricto | 0.45 | 0.78 | 0.81 | 1.25 | 0.33 | 0.59 | 0.65 | 1.20 | 0.69 | 0.56 | 0.61 | 1.31 | 0.81 | 1.04 | 1.43 | 2.41 | 0.211 | 0.155 | 0.3 | <0.001 | 0.129 | 0.151 | 0.131 | 0.202 |
|  | Clostridiales_Incertae Sedis XIII |  | 1.72 | 1.88 | 1.80 | 2.45 | 1.57 | 1.73 | 1.70 | 2.23 | 2.02 | 2.11 | 2.16 | 2.52 | 1.80 | 1.81 | 1.76 | 2.50 | 0.081 | 0.005 | 0.006 | <0.001 | 0.468 | 0.484 | 0.4 | 0.049 |
|  |  | Anaerovorax | 1.45 | 1.54 | 1.50 | 1.70 | 1.29 | 1.46 | 1.42 | 1.53 | 1.58 | 1.63 | 1.64 | 1.79 | 1.38 | 1.25 | 1.32 | 1.72 | 0.077 | 0.483 | 0.033 | <0.001 | 0.418 | 0.065 | 0.567 | 0.063 |
|  |  | Mogibacterium | 1.31 | 1.53 | 1.40 | 2.28 | 1.24 | 1.35 | 1.25 | 2.04 | 1.80 | 1.89 | 1.91 | 2.35 | 1.57 | 1.63 | 1.52 | 2.35 | 0.117 | 0.001 | 0.024 | <0.001 | 0.662 | 0.259 | 0.496 | 0.208 |
|  | Eubacteriaceae |  | 1.29 | 1.46 | 1.27 | 1.40 | 1.38 | 1.25 | 1.08 | 1.35 | 0.93 | 0.90 | 0.88 | 1.13 | 0.76 | 0.88 | 0.69 | 1.04 | 0.135 | 0.041 | 0.562 | 0.017 | 0.937 | 0.416 | 0.644 | 0.409 |
|  |  | Eubacterium | 1.01 | 1.01 | 0.91 | 1.13 | 1.20 | 0.81 | 0.66 | 1.11 | 0.88 | 0.68 | 0.74 | 1.08 | 0.72 | 0.75 | 0.63 | 0.92 | 0.159 | 0.245 | 0.597 | 0.003 | 0.939 | 0.566 | 0.629 | 0.232 |
|  |  | Pseudoramibacter | 1.00 | 1.30 | 1.05 | 1.09 | 0.95 | 1.07 | 0.93 | 0.98 | 0.27 | 0.56 | 0.50 | 0.30 | 0.25 | 0.44 | 0.23 | 0.34 | 0.119 | 0.005 | 0.55 | 0.024 | 0.921 | 0.704 | 0.343 | 0.501 |
|  | Lachnospiraceae |  | 3.32 | 3.24 | 3.35 | 3.09 | 3.40 | 3.23 | 3.33 | 3.13 | 3.14 | 3.12 | 3.24 | 3.06 | 3.10 | 3.12 | 3.28 | 2.98 | 0.075 | 0.004 | 0.933 | <0.001 | 0.591 | 0.168 | 0.926 | 0.511 |
|  |  | Blautia | 0.84 | 0.83 | 0.58 | 0.86 | 0.91 | 0.73 | 0.73 | 0.94 | 1.09 | 0.86 | 0.77 | 0.93 | 0.88 | 0.88 | 0.80 | 0.93 | 0.122 | 0.264 | 0.959 | 0.01 | 0.556 | 0.823 | 0.501 | 0.408 |
|  |  | Butyrivibrio | 2.54 | 2.38 | 2.29 | 2.18 | 2.50 | 2.24 | 2.25 | 2.17 | 2.36 | 2.18 | 2.08 | 2.05 | 2.28 | 2.16 | 2.18 | 2.03 | 0.076 | 0.036 | 0.61 | <0.001 | 0.658 | 0.725 | 0.425 | 0.486 |
|  |  | Clostridium XlVa | 1.70 | 2.05 | 2.10 | 1.42 | 1.98 | 2.17 | 2.20 | 1.61 | 1.84 | 2.17 | 2.33 | 1.80 | 2.10 | 2.41 | 2.70 | 1.98 | 0.076 | 0.003 | 0.008 | <0.001 | 0.494 | 0.006 | 0.608 | 0.231 |
|  |  | Coprococcus | 1.21 | 1.25 | 1.52 | 1.13 | 1.43 | 1.29 | 1.45 | 1.20 | 1.36 | 1.30 | 1.43 | 1.21 | 1.16 | 1.08 | 1.48 | 1.18 | 0.122 | 0.818 | 0.896 | 0.006 | 0.582 | 0.682 | 0.639 | 0.209 |
|  |  | Lachnobacterium | 1.10 | 0.99 | 1.20 | 1.27 | 1.01 | 0.95 | 1.16 | 1.34 | 0.85 | 0.74 | 0.71 | 0.89 | 0.83 | 0.62 | 0.79 | 0.84 | 0.117 | <0.001 | 0.679 | 0.006 | 0.997 | 0.197 | 0.708 | 0.629 |
|  |  | Lachnospiracea_incertae_sedis | 2.43 | 2.32 | 2.32 | 2.32 | 2.50 | 2.33 | 2.24 | 2.46 | 1.95 | 1.77 | 1.71 | 2.07 | 1.90 | 1.88 | 1.81 | 1.96 | 0.104 | <0.001 | 0.642 | 0.014 | 0.807 | 0.342 | 0.85 | 0.189 |
|  |  | Oribacterium | 0.61 | 0.55 | 0.42 | 0.44 | 0.76 | 0.54 | 0.43 | 0.58 | 1.09 | 0.82 | 0.65 | 1.10 | 0.86 | 0.83 | 0.68 | 0.94 | 0.139 | <0.001 | 0.918 | 0.013 | 0.205 | 0.249 | 0.893 | 0.327 |
|  |  | Pseudobutyrivibrio | 2.23 | 2.31 | 2.41 | 2.09 | 2.19 | 2.13 | 2.34 | 2.09 | 2.13 | 2.12 | 2.24 | 2.10 | 2.02 | 2.16 | 2.35 | 2.09 | 0.087 | 0.181 | 0.548 | <0.001 | 0.478 | 0.395 | 0.536 | 0.301 |
|  |  | Roseburia | 2.31 | 2.26 | 2.82 | 1.56 | 2.39 | 2.23 | 2.69 | 1.90 | 2.15 | 2.30 | 2.73 | 1.82 | 2.06 | 2.11 | 2.54 | 1.53 | 0.169 | 0.265 | 0.536 | <0.001 | 0.215 | 0.571 | 0.587 | 0.346 |
|  | Lactobacillaceae | Lactobacillus | 2.94 | 3.04 | 2.78 | 2.56 | 3.01 | 3.00 | 2.70 | 2.49 | 2.57 | 2.49 | 2.59 | 2.05 | 2.53 | 2.54 | 2.41 | 2.21 | 0.160 | 0.023 | 0.906 | 0.002 | 0.918 | 0.369 | 0.554 | 0.506 |
|  | Peptococcaceae 1 |  | 1.78 | 1.75 | 1.71 | 2.41 | 1.45 | 1.37 | 1.48 | 2.19 | 2.00 | 2.03 | 2.11 | 2.35 | 1.69 | 1.59 | 1.65 | 2.24 | 0.087 | 0.071 | 0.009 | <0.001 | 0.851 | 0.029 | 0.093 | 0.238 |
|  |  | Dehalobacter | 1.61 | 1.58 | 1.52 | 2.16 | 1.29 | 1.18 | 1.26 | 1.88 | 1.81 | 1.81 | 1.88 | 2.09 | 1.46 | 1.35 | 1.37 | 1.92 | 0.095 | 0.168 | 0.008 | <0.001 | 0.79 | 0.092 | 0.192 | 0.334 |
|  | Ruminococcaceae |  | 3.07 | 3.06 | 2.95 | 3.22 | 2.92 | 2.85 | 2.77 | 3.04 | 3.40 | 3.29 | 3.09 | 3.22 | 3.31 | 3.17 | 2.99 | 3.25 | 0.067 | <0.001 | 0.014 | <0.001 | 0.219 | 0.014 | 0.508 | 0.589 |
|  |  | Acetanaerobacterium | 1.07 | 0.87 | 0.62 | 0.44 | 0.86 | 0.77 | 0.51 | 0.31 | 1.10 | 1.01 | 0.85 | 0.54 | 0.65 | 0.68 | 0.61 | 0.54 | 0.189 | 0.63 | 0.173 | <0.001 | 0.673 | 0.429 | 0.528 | 0.677 |
|  |  | Acetivibrio | 1.87 | 2.06 | 2.03 | 2.39 | 1.97 | 2.08 | 2.06 | 2.22 | 2.01 | 2.10 | 2.15 | 2.23 | 1.90 | 2.05 | 2.08 | 2.27 | 0.079 | 0.79 | 0.639 | <0.001 | 0.668 | 0.425 | 0.794 | 0.093 |
|  |  | Clostridium IV | 2.20 | 2.20 | 2.08 | 2.51 | 2.01 | 1.96 | 1.81 | 2.37 | 2.52 | 2.52 | 2.39 | 2.44 | 2.15 | 2.12 | 2.01 | 2.42 | 0.120 | 0.044 | 0.009 | <0.001 | 0.586 | 0.148 | 0.182 | 0.515 |
|  |  | Ethanoligenens | 0.57 | 0.35 | 0.50 | 0.90 | 0.55 | 0.51 | 0.48 | 1.16 | 0.94 | 1.04 | 1.01 | 1.49 | 0.92 | 1.15 | 1.18 | 1.31 | 0.132 | 0.024 | 0.757 | <0.001 | 0.839 | 0.065 | 0.639 | 0.148 |
|  |  | Flavonifractor | 0.08 | 0.15 | 0.15 | 0.44 | 0.08 | 0.00 | 0.08 | 0.23 | 0.29 | 0.25 | 0.38 | 0.25 | 0.08 | 0.08 | 0.23 | 0.12 | 0.234 | 0.497 | 0.125 | 0.103 | 0.736 | 0.067 | 0.888 | 0.613 |
|  |  | Oscillibacter | 2.18 | 2.27 | 2.20 | 2.23 | 1.81 | 1.95 | 1.83 | 1.67 | 2.09 | 2.10 | 2.14 | 1.83 | 2.22 | 2.16 | 2.25 | 1.63 | 0.223 | 0.818 | 0.253 | 0.109 | 0.195 | 0.265 | 0.455 | 0.869 |
|  |  | Ruminococcus | 2.72 | 2.55 | 2.24 | 2.27 | 2.62 | 2.20 | 2.03 | 2.17 | 3.19 | 2.90 | 2.30 | 2.61 | 3.13 | 2.84 | 2.32 | 2.80 | 0.111 | 0.002 | 0.418 | <0.001 | 0.323 | 0.042 | 0.206 | 0.558 |
|  | Streptococcaceae | Streptococcus | 2.24 | 2.73 | 2.62 | 2.96 | 2.51 | 2.93 | 2.79 | 3.32 | 1.94 | 2.17 | 2.25 | 2.70 | 2.02 | 2.29 | 2.32 | 2.83 | 0.121 | 0.013 | 0.258 | <0.001 | 0.622 | 0.257 | 0.659 | 0.817 |
|  | Veillonellaceae |  | 2.58 | 2.63 | 2.59 | 2.30 | 2.62 | 2.63 | 2.60 | 2.28 | 2.67 | 2.70 | 2.75 | 2.56 | 2.69 | 2.77 | 2.80 | 2.71 | 0.098 | 0.033 | 0.585 | <0.001 | 0.669 | 0.067 | 0.955 | 0.718 |
|  |  | Anaerovibrio | 1.38 | 1.56 | 1.65 | 1.41 | 1.63 | 1.71 | 1.76 | 1.60 | 1.43 | 1.42 | 1.43 | 1.41 | 1.41 | 1.59 | 1.59 | 1.52 | 0.085 | 0.223 | 0.149 | 0.01 | 0.704 | 0.318 | 0.915 | 0.27 |
|  |  | Selenomonas | 2.48 | 2.55 | 2.49 | 2.06 | 2.53 | 2.54 | 2.49 | 2.08 | 2.55 | 2.61 | 2.66 | 2.33 | 2.57 | 2.67 | 2.70 | 2.54 | 0.103 | 0.054 | 0.551 | <0.001 | 0.697 | 0.038 | 0.726 | 0.688 |
| Proteobacteria |  |  | 1.13 | 1.26 | 1.29 | 1.62 | 1.01 | 1.11 | 0.99 | 1.36 | 1.25 | 1.39 | 1.49 | 1.81 | 1.25 | 1.45 | 1.48 | 1.81 | 0.106 | 0.007 | 0.244 | <0.001 | 0.199 | 0.378 | 0.627 | 0.833 |
|  | Comamonadaceae |  | 0.15 | 0.23 | 0.23 | 0.00 | 0.27 | 0.23 | 0.00 | 0.08 | 0.08 | 0.00 | 0.00 | 0.00 | 0.08 | 0.00 | 0.00 | 0.00 | 0.107 | 0.005 | 0.913 | 0.149 | 0.913 | 0.355 | 0.361 | 0.361 |
|  | Oxalobacteraceae |  | 0.00 | 0.24 | 0.00 | 0.08 | 0.00 | 0.00 | 0.00 | 0.00 | 0.00 | 0.00 | 0.08 | 0.00 | 0.00 | 0.12 | 0.00 | 0.00 | 0.104 | 0.68 | 0.36 | 0.314 | 0.232 | 0.613 | 0.791 | 0.222 |
|  | Pseudomonadaceae |  | 0.08 | 0.27 | 0.08 | 0.08 | 0.15 | 0.08 | 0.00 | 0.00 | 0.19 | 0.08 | 0.15 | 0.23 | 0.15 | 0.12 | 0.08 | 0.19 | 0.154 | 0.313 | 0.409 | 0.726 | 0.716 | 0.424 | 0.847 | 0.623 |
|  | Sutterellaceae | Sutterella | 0.49 | 0.67 | 0.80 | 0.86 | 0.35 | 0.65 | 0.64 | 0.58 | 0.69 | 1.09 | 1.15 | 1.22 | 0.84 | 1.23 | 1.20 | 1.41 | 0.132 | <0.001 | 0.917 | <0.001 | 0.181 | 0.318 | 0.75 | 0.635 |
| Spirochaetes | Spirochaetaceae |  | 0.23 | 0.27 | 0.00 | 0.42 | 0.19 | 0.00 | 0.08 | 0.19 | 0.77 | 0.56 | 0.63 | 0.81 | 0.41 | 0.31 | 0.35 | 0.66 | 0.171 | <0.001 | 0.019 | 0.036 | 0.285 | 0.748 | 0.76 | 0.426 |
|  |  | Treponema | 0.15 | 0.25 | 0.00 | 0.31 | 0.19 | 0.00 | 0.08 | 0.12 | 0.69 | 0.42 | 0.46 | 0.63 | 0.34 | 0.29 | 0.31 | 0.36 | 0.156 | 0.001 | 0.045 | 0.209 | 0.302 | 0.763 | 0.563 | 0.376 |
| Tenericutes | Acholeplasmataceae |  | 0.23 | 0.27 | 0.08 | 0.35 | 0.08 | 0.08 | 0.08 | 0.08 | 0.27 | 0.27 | 0.19 | 0.19 | 0.17 | 0.24 | 0.19 | 0.08 | 0.134 | 0.385 | 0.078 | 0.638 | 0.415 | 0.459 | 0.503 | 0.85 |
|  |  | Acholeplasma | 0.00 | 0.08 | 0.00 | 0.08 | 0.00 | 0.00 | 0.00 | 0.00 | 0.00 | 0.08 | 0.08 | 0.00 | 0.08 | 0.08 | 0.00 | 0.08 | 0.062 | 0.419 | 0.784 | 0.535 | 0.419 | 0.857 | 0.535 | 0.332 |
|  |  | Anaeroplasma | 0.23 | 0.24 | 0.08 | 0.27 | 0.08 | 0.08 | 0.08 | 0.08 | 0.27 | 0.23 | 0.15 | 0.19 | 0.15 | 0.19 | 0.19 | 0.00 | 0.134 | 0.428 | 0.034 | 0.699 | 0.539 | 0.568 | 0.42 | 0.943 |
| Verrucomicrobia | Subdivision5_genera_incertae_sedis |  | 1.29 | 1.38 | 1.39 | 1.80 | 1.08 | 1.26 | 1.29 | 1.63 | 1.69 | 1.96 | 1.94 | 1.94 | 1.65 | 1.86 | 1.94 | 1.92 | 0.119 | 0.001 | 0.393 | <0.001 | 0.598 | 0.013 | 0.905 | 0.884 |
| Unclassified |  |  | 0.44 | 0.46 | 0.48 | 0.93 | 0.30 | 0.00 | 0.42 | 0.89 | 0.63 | 0.44 | 0.48 | 1.20 | 0.39 | 0.23 | 0.44 | 0.84 | 0.164 | 0.147 | 0.008 | <0.001 | 0.748 | 0.792 | 0.351 | 0.366 |

^1^Standard error of the difference for the interaction between the type of forage (F). vitamin E supplementation at 50 IU/d (V) and incubation time (T) (n=4). The total number of reads per sample was log-transformed and minor genera (<0.01%) were discarded.

**REFERENCES**

Bayer K, Kamke J & Hentschel U (2014) Quantification of bacterial and archaeal symbionts in high and low microbial abundance sponges using real-time PCR. *Fems Microbiol. Ecol.* **89**: 679-690.

Denman SE & McSweeney CS (2006) Development of a real-time PCR assay for monitoring anaerobic fungal and cellulolytic bacterial populations within the rumen. *FEMS Microbiol. Ecol.* **58**: 572-582.

Denman SE, Tomkins N & McSweeney CS (2007) Quantitation and diversity analysis of ruminal methanogenic populations in response to the antimethanogenic compound bromochloromethane. *Fems Microbiology Ecology* **62**: 313-322.

Edwards JE, Huws SA, Kim EJ & Kingston-Smith AH (2007) Characterization of the dynamics of initial bacterial colonization of nonconserved forage in the bovine rumen. *FEMS Microbiol Ecol.* **62**: 323-335.

Maeda H, Fujimoto C, Haruki Y, Maeda T, Kokeguchi S, Petelin M, Arai H, Tanimoto I, Nishimura F & Takashiba S (2003) Quantitative real-time PCR using TaqMan and SYBR Green for *Actinobacillus actinomycetemcomitans , Porphyromonas gingivalis , Prevotella intermedia* , tetQ gene and total bacteria. *FEMS Immunol. Med. Microbiol.* **39**: 81 – 86.

Sylvester JT, Karnati SKR, Yu ZT, Morrison M & Firkins JL (2004) Development of an assay to quantify rumen ciliate protozoal biomass in cows using real-time PCR. *J. Nutr.* **134**: 3378-3384
